# Dual repression modes triggered by constitutively stabilized beta-catenin in the dorsal neural tube underlie cell fate alterations of the roof plate, neural crest, and dorsal interneurons

**DOI:** 10.1186/s13578-026-01615-x

**Published:** 2026-06-30

**Authors:** Subbroto Kumar Saha, Tianyu Zhao, Kurt Reynolds, Moira McMahon, Chengji J. Zhou

**Affiliations:** 1 ADA Forsyth Institute, 100 Chestnut Street, Somerville, MA 02143 USA; 2 Institute for Pediatric Regenerative Medicine, Shriners Hospitals for Children- Northern California, School of Medicine, University of California at Davis, Sacramento, CA 95817 USA; 3 School of Life Sciences, Fudan University, Shanghai, 200438 China

**Keywords:** Roof plate, Cell fate determination, Neural crest cell differentiation, Dorsal neural tube patterning, Wnt/ß-catenin signaling, Transcriptional repression, Negative feedback loop

## Abstract

The roof plate that covers the dorsal neural tube is a key organizer or signaling center that generates morphogenetic signaling proteins to regulate neural crest cell production and dorsal CNS patterning. However, knowledge remains limited regarding the molecular mechanism regulating roof plate cell fate and function. Loss of function of Wnt/ß-catenin signaling in the roof plate or dorsal neural folds can prevent neural tube closure, neural crest cell production, and dorsal CNS patterning. In this study, we carried out conditional activation of ß-catenin in the dorsal neural folds of mice and found dramatic cell fate alterations, which include anteriorly expressed *Cdx2* of the caudal-type homeobox gene in the dorsal neural tube, swapped expression patterns of the roof plate markers *Bmp6* and *Lmx1a* with their respective homologues *Bmp4* and *Lmx1b*, ectopically expressed *Msx1* of the neural crest inducer in the migratory stream and dorsal root ganglia, and completely abolished expression of the neural crest cell differentiation marker *Sox10* and the dorsal spinal interneuron precursor marker *Atoh1*. Our in vitro studies using a neural crest cell line demonstrate that the full-length ß-catenin, not the signaling defective ß-catenin significantly upregulates *Msx1* and represses *Sox10* and *Atoh1* expression, which can be rescued by *Msx1* knockdown, supporting an indirectly repressive role of the stabilized ß-catenin acting through Msx1 on *Sox10* and *Atoh1* expression. Intriguingly, *Wnt1* and *Wnt3a* are drastically abolished in the roof plate of the ß-catenin stabilized embryos. However, *Msx1* knockdown does not upregulate or rescue *Wnt1* and *Wnt3a* expression when they are repressed by ß-catenin in vitro. Further in vitro studies reveal a direct repression of *Wnt1* and *Wnt3a* by ß-catenin transcriptional complex, implicating a novel negative feedback loop of Wnt/ß-catenin signaling pathway at the ligand level. Together, these results suggest that constitutively stabilized ß-catenin triggers dual repression modes to prevent specification and differentiation of multiple cell lineages derived from the dorsal neural tube, but it may not prevent the presumptively undifferentiated *Msx1*-expressing neural crest cell precursors migrating out the dorsal neural tube to form the dorsal root ganglia, and that a finely tuned Wnt/ß-catenin signaling level is crucial for proper cell fate determination and functional modulation of the dorsal neural tube and its derivatives.

**Supplementary Information:**

The online version contains supplementary material available at https://doi.org/10.1186/s13578-026-01615-x.

## Introduction

During mammalian neurulation, the dorsal tips of the bilateral neural folds approach each other and fuse at the dorsal midline, which is guided by the dynamic actions of multicellular rosette formation and F-actin-based convergent protrusions of the non-neural surface ectodermal cells surrounding the closure site [1]. The fused non-neural ectodermal cells are likely removed by programmed cell death, then the adjacent neuroepithelial cells become confluent and form the roof plate covering the dorsal neural tube [2, 3]. The precursor roof plate (and the dorsal edge of the non-neural ectoderm of the neural fold) is well known for its crucial function acting as a regional organizer or signaling center, which secretes several key morphogenetic proteins, such as Bmps and Wnts, to regulate neural crest cell productions and dorsal CNS patterning [4–9]. Bone morphogenetic proteins (Bmps) secreted from the adjacent surface ectoderm may play an inductive role in roof plate formation [4, 10]. Ectopic activation of Bmp signaling is sufficient to induce the roof plate cell fate in chicken embryos. Conversely, Bmp inhibitors can cause a defective roof plate formation [11]. At the transcription factor level, studies on the spontaneous mutant Dreher mice identified Lmx1a as a key factor for roof plate development [12]. Lmx1a is a LIM domain transcription factor and is expressed in the spinal roof plate posterior to the brain region and it has been identified as mediator of BMP signaling [11]. The close homologue Lmx1b also plays important role in roof plate formation by regulating roof plate progenitors and differentiated roof plate cells [13, 14].

Wnt signaling plays crucial roles in development and disease. Among 19 Wnt ligands, Wnt1 and Wnt3a are exclusively expressed in the roof plate and play synergetic roles in neural crest production, somite patterning, and dorsal spinal cord patterning [5, 7, 15]. In the canonical Wnt signaling pathway, Wnt proteins concurrently bind to two distinct types of receptors, the single-transmembrane receptor Lrp6 or Lrp5, and a seven-pass transmembrane Fzd receptor, which triggers a series of intracellular signaling molecule actions, resulting in the stabilization of the cytosolic ß-catenin that translocates into the nucleus and binds to Tcf/Lef1 transcriptional complex and activate various downstream target genes [16–18]. It has been demonstrated that ß-catenin regulates neural progenitor pools and subsequently the size of the brain and spinal cord [19–21]. We have previously demonstrated that conditional ablation of either ß-catenin (encoded by *Ctnnb1*) or Lrp6 in the Pax3-expressing dorsal neural folds and subsequent roof plate causes spinal neural tube closure defects [22, 23], and that conditional activation of ß-catenin can prevent neural tube defects in Lrp6 mutants [23]. However, we did not observe significant changes of the roof plate marker genes in the Wnt/ß-catenin signaling loss-of-function mutants. Nevertheless, a recent study demonstrates that Wnt/ß-catenin signaling is required for apical constriction of the roof plate cells [24].

In the current study, we addressed the effects of gain-of-function (GOF) of ß-catenin signaling in the roof plate formation and modulation. Conditional removal of the floxed *Ctnnb1* exon 3 that encodes a phosphorylation site for ß-catenin protein degradation will stabilize ß-catenin [25], leading to constitutive activation of canonical Wnt signaling in the Cre-expressing lineage cells. Our results revealed dramatic alterations of the roof plate cell fate and morphology in the conditional *Ctnnb1*-GOF mutants, including switched expression patterns of a roof plate marker gene *Lmx1a* with its homologue *Lmx1b*, switched *Bmp6* with *Bmp4*, and ectopic expression of a caudal-type homeobox gene *Cdx2* in more anterior regions of spinal roof plate. Consequently, constitutively stabilized ß-catenin in the dorsal neural tube attenuates interneuron specification in the dorsal spinal cord and neural crest cell differentiation in the migratory stream and dorsal root ganglia. In contrast to the well-established transcriptional activation of the Wnt/ß-catenin signaling downstream target genes, the current study reveals novel molecular mechanisms underlying the stabilized ß-catenin that triggers two distinctly different repression modes to alter the cell fate of the roof plate and its associated structures during vertebrate neural tube development.

## Materials and methods

### Mice

**Fig. 1:**
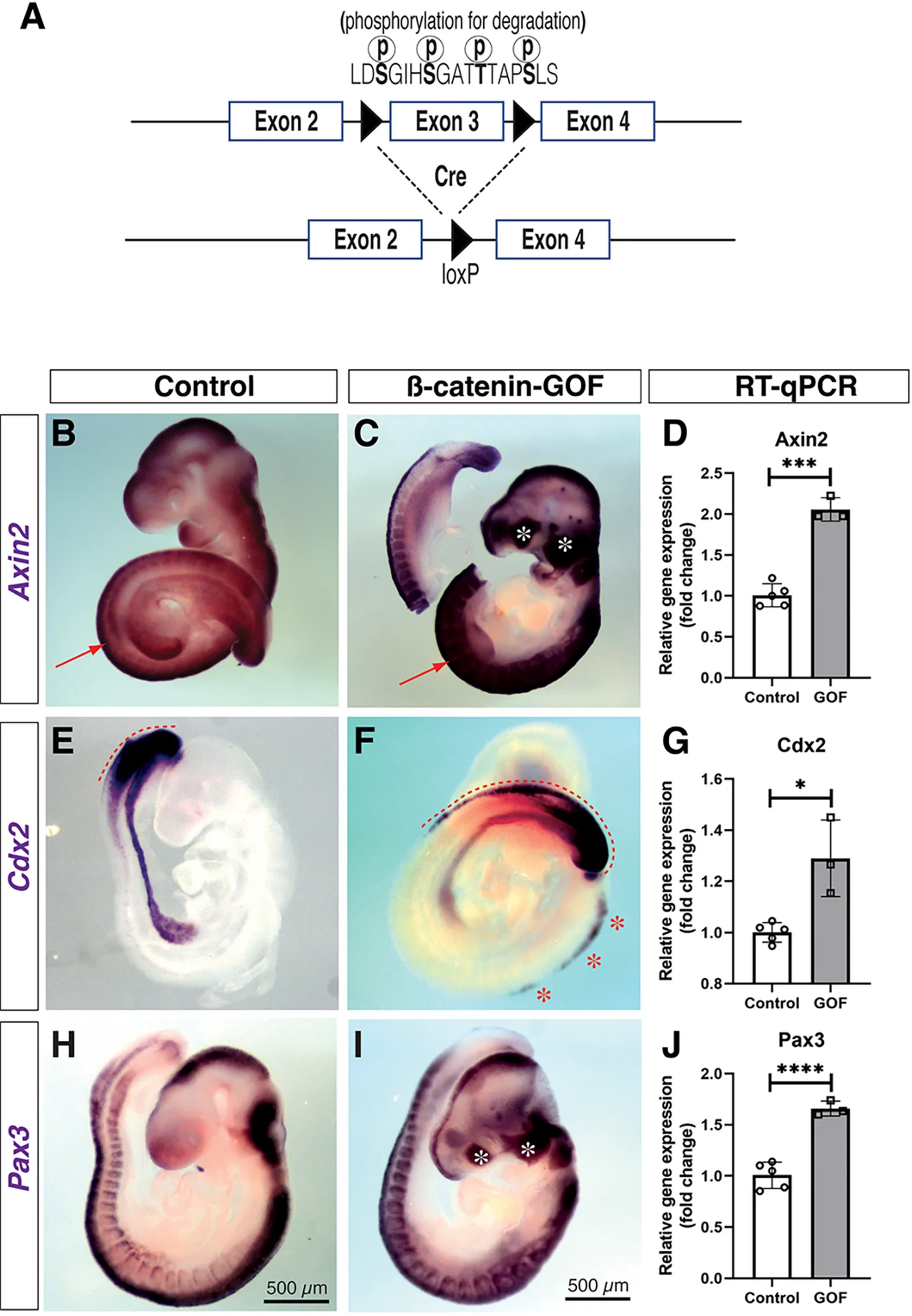
Constitutive activation of ß-catenin in Pax3-expressing lineage cells causes significant upregulation and ectopic expression of representative downstream target genes in E9.5 mouse embryos. **A **Schematic illustration of the conditional gain-of-function (GOF) of ß-catenin mouse model. The loxP-flanked Ctnnb1 exon 3 encodes crucial serine/threonine phosphorylation sites by CK1a and Gsk3ß in the ß-catenin destruction complex. In frame excision of the exon 3 via Cre-loxP recombination allows the cell to generate the shortened and constitutively stabilized ß-catenin protein. **B**-**D ***Axin2* expression is markedly upregulated in the mutant neural tube (arrows in **C**) and ectopically expanded in the facial region (asterisks in **C**) demonstrated by wholemount in situ hybridization (**B**, **C**) and confirmed by RT-qPCR (**D**). **E**-**G ***Cdx2* ectopically appeared at the thoracic region (asterisks in **F**) and anteriorly expanded in the caudal neural tube (dashed curve in **F**) of the mutant embryos. **H**-**J** *Pax3* is moderately upregulated in the mutant neural tube and somites, with notably ectopic expression in the mutant facial regions (asterisks in **I**). E9.5 whole embryos were used for RT-qPCR: Control *n* = 5, GOF *n* = 3. *, *P* < 0.05; ***, *P* < 0.001; ****, *P* < 0.0001

*Pax3*^*Cre/+*^ knockin mice (Jackson Laboratory strain # 005549, donated by J Epstein, University of Pennsylvania) [26] were mated with *Ctnnb1*^*flox(ex3)*^ mice (MGI:1858008, gift of M. Taketo, Kyoto University, Japan) [25] to generate the conditional ß-catenin gain-of-function embryos for the study (Fig. 1A). Pregnant, timed-mated mice were euthanized prior to cesarean section. The noon of the conception day was designated as E0.5. The animal protocol was approved by Institutional Animal Care and Use Committee.

### Wholemount in situ hybridization

Wholemount in situ hybridization was performed as previously described [22]. Briefly, embryos were fixed in 4% PFA overnight in cold room and proceeded for wholemount in situ hybridization using digoxigenin-labeled antisense RNA probes that were synthesized based on sequence information provided by Allen Brain Atlas. Selected embryos after color development were embedded in O.C.T. compound and sectioned by a cryostat at 20 μm for microscopic imaging. All in situ experiments were triplicated with consistent results per probe.

### Embryonic RNA isolation and RT-qPCR

Total RNAs were isolated from E9.5 whole embryos and E10.5 embryos excluding heads. The non-Cre embryos were used as the control. After reverse transcription (RT), real-time quantitative PCR (qPCR) was carried out as described [27]. The mRNA levels of the target genes were normalized to that of the house-keeping gene *Gapdh* to allow for comparisons between the control and mutant embryos using the ΔCT method. 5 controls and 3 mutant embryos at E9.5 and 4 controls and 4 mutants at E10.5 were analyzed. Student’s t-test was conducted and *P* < 0.05 is considered statistically significant. Primer sequences are listed in the supplementary Table S1.

### Cell culture, ß-catenin signaling modulation, and Msx1 knockdown experiments

The O9-1 mouse cranial neural crest cells (Sigma-Aldrich, SCC049) were cultured according to the published protocol [28]. Transfection was performed using Lipofectamine 3000 (Invitrogen) or the siRNA transfection reagent (Roche) according to the manufacturers’ instructions. For ß-catenin signaling modulation, the cells were transected with 5 µg of either full-length ß-catenin (ß-cat-FL) or double signaling mutant ß-catenin-dm expression plasmids. The empty vector pcDNA served as the control. For siRNA knockdown, the cells were transfected with TriFECTa DsiRNA (Integrated DNA Technologies) targeting *Msx1* at a final concentration of 10 nM. Scramble siRNA was used as the control. 48 h after transfection, total RNA was extracted for RT-qPCR. All experiments were performed in triplicate. Primer sequences are listed in the supplementary Table S1.

### Chromatin immunoprecipitation (ChIP) assay

ChIP assay was carried out as previously described [22, 27]. Chromatin extracts were prepared from wild-type embryos. Anti-Msx1 (ThermoFisher Scientific, H00004487-M11) and anti-ß-catenin antibodies (Santa Cruz Biotech, sc-7199) were used to pull down respective transcriptional DNA binding complexes and rabbit IgG (Invitrogen; 10500 C) was used for the negative control. The activation sites were amplified with specific primers.

### Luciferase reporter assay

For Msx1 reporter assays, presumptive regulatory regions of *Sox10* (BS3), *Atoh1* (BS4), *Wnt1* (BS5), and *Wnt3a* (BS1) were PCR-amplified and cloned into the pGL4-basic luciferase reporter vector (Promega). Mutant promoter constructs were generated by PCR-based site-directed mutagenesis [29]. O9-1 cells were co-transfected with either wild-type or mutant promoter–luciferase reporters (1 µg) and graded amounts (40, 80, 160, or 320 ng) of an Msx1 expression plasmid (pRP[Exp]-Puro-EF1A>3xFLAG/mMsx1; VectorBuilder). A pGL4-basic luciferase reporter vector or pRP[Exp]-Puro-EF1A empty vector served as the control. Luciferase activity was measured 24 h after transfection using the Dual-Luciferase Reporter Assay System (Promega) following the manufacturer’s protocol. For ß-catenin reporter assays, the candidate repression motif *AGAAAA* was found in *Wnt1* downstream and *AGATAA* was found in *Wnt3a* intron 1. The “wild-type” or “mutated” repression motifs were amplified by PCR and cloned into the pGL2-basic luciferase reporter vector. Similar to previously described studies [22, 27], L cells were transiently transfected with the wild-type or mutated motif-driven luciferase reporters, together with the vector controls or the constitutively active ß-catenin constructs [30]. 24 h after transfection, luciferase activities were measured using the Dual-Luciferase Assay Kit.

## Results

### Upregulation and ectopic expression of canonical Wnt signaling downstream target genes in the ß-catenin stabilized mutants

ß-catenin (encoded by *Ctnnb1* gene) is the key intracellular signaling transducer in the canonical Wnt pathway and plays crucial roles in development and disease, mostly acting through transcriptional activation of various downstream target genes [31]. We previously found that loss-of-function (LOF) of canonical Wnt signaling by conditional ablation of ß-catenin (through mating *Ctnnb1*^*flox(ex2–6)*^ mice [32] with *Pax3*^*Cre*^ knock in mice [26]) causes spinal neural tube closure defects by downregulating key transcriptional target genes including *Cdx2* and *Pax3* in the dorsal neural folds during neurulation [22]. To address the effects of gain-of-function of canonical Wnt signaling in neural tube development in the current study, *Ctnnb1*^*flox(ex3)*^ mice [25] were mated with *Pax3*^*Cre*^ knockin mice, in which conditional removal of the floxed *Ctnnb1* (encodes ß-catenin) exon 3 that encodes phosphorylation sites for ß-catenin protein degradation will stabilize ß-catenin (Fig. 1A), leading to constitutive activation of canonical Wnt signaling in the Pax3-expressing lineage cells. As expected, ß-catenin stabilization significantly upregulates *Axin2*, a general downstream target and a negative feedback regulator in the canonical Wnt pathway [33] in the *Pax3*-expressing lineage cells, including dorsal neuroepithelial cells and roof plate, neural crest cells, and somites at E9.5, demonstrated by both wholemount in situ hybridization and RT-qPCR (Fig. 1B-D). Notably, *Axin2* is ectopically upregulated in the craniofacial region (mainly trigeminal ganglia) of the ß-catenin-GOF mutants (Fig. 1C). Nevertheless, neural tube closure in the ß-catenin-GOF mutants proceeds with similar timing as littermate controls. In contrast to the diminished expression of *Cdx2* and *Pax3* in the ß-catenin-LOF mutants as demonstrated in our previous publication [22], these two target genes are significantly upregulated in the ß-catenin-GOF embryos (Fig. 1E-J). The expression domain of the caudal-type homeobox gene *Cdx2* in the dorsal midline or roof plate is significantly extended anteriorly in the ß-catenin-GOF mutant embryos at E9.5 with ectopic expression in the thoracic roof plate as well (Fig. 1F). *Pax3* is upregulated in multiple expression sites with ectopic expression in the craniofacial region (Fig. 1I), which is nearly identical to *Axin2* upregulation patterns in the ß-catenin-GOF mutants. These results indicate a consistent role of ß-catenin in transcriptional activation of the representative downstream target genes during neurulation and related developmental processes.

### Ectopic expression of *Msx1* in the neural crest migratory stream and dorsal root ganglia and attenuated neural crest cell differentiation in ß-catenin stabilized mutants

**Fig. 2:**
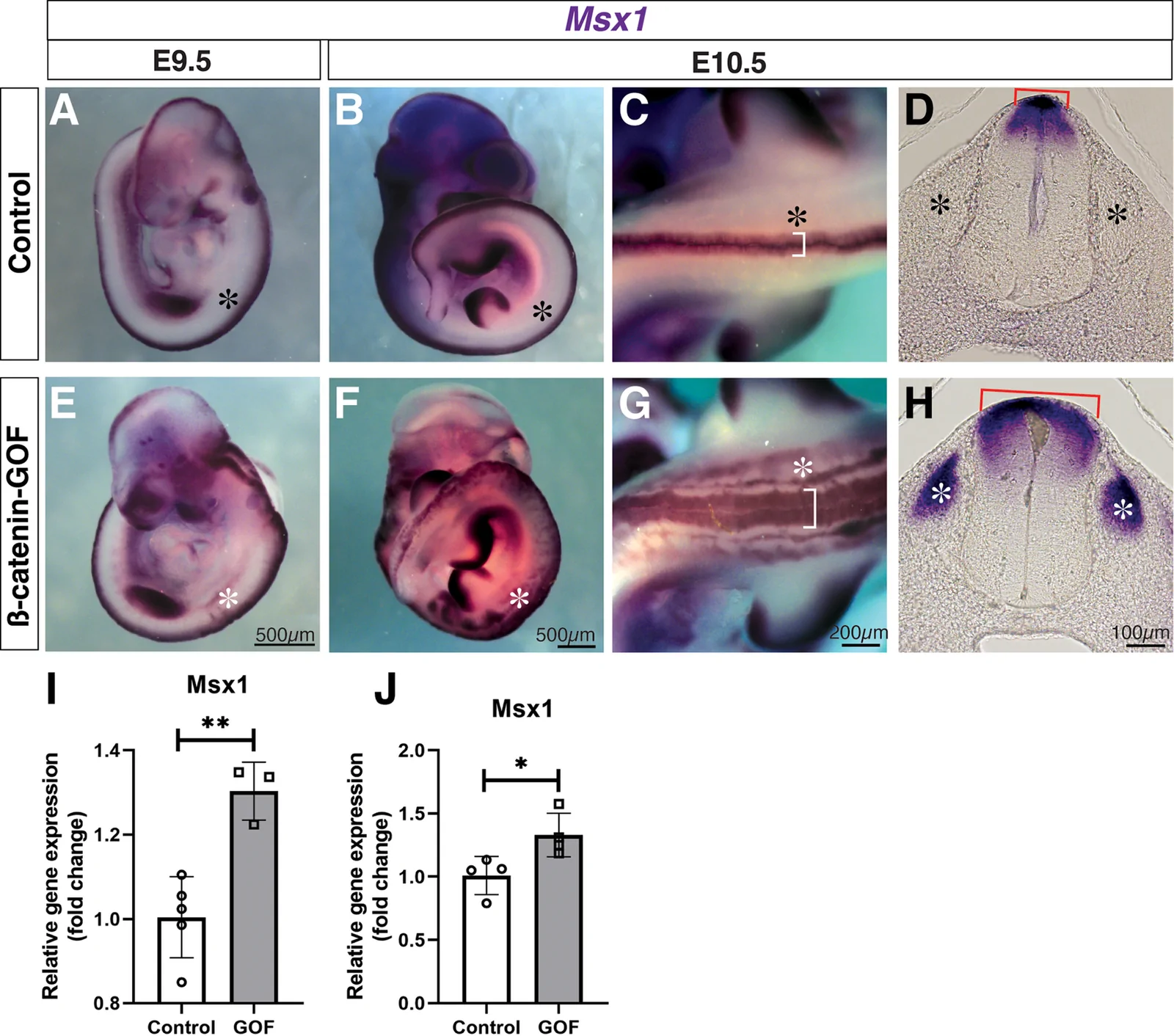
Significant expansion and ectopic expression of *Msx1* in the ß-catenin-GOF mutants. **A**-**H **Wholemount in situ hybridization of *Msx1* demonstrates ectopic expression in the migratory neural crest cells at the thoracic region of the E9.5 mutant (asterisk in **E**) and in the dorsal root ganglia at E10.5 (asterisks in **F**-**H**). The *Msx1* expression domain in the roof plate is significantly widened in the mutant compared to the control at E10.5 (brackets in **C**, **D**,**G**, **H**). **A**, **B**, **E**, **F**, lateral views of whole mouse embryos; **C**,**G**, dorsal spine views of E10.5 embryos; **D**,**H**, transverse sections at the thoracic spine level. **I**, **J **RT-qPCR results of *Msx1* at E9.5 (**I**, 5 controls and 3 mutants of the whole embryos) and E10.5 (**J**, 4 controls and 4 mutants without heads); *, *P* < 0.05; **, *P* < 0.01

**Fig. 3:**
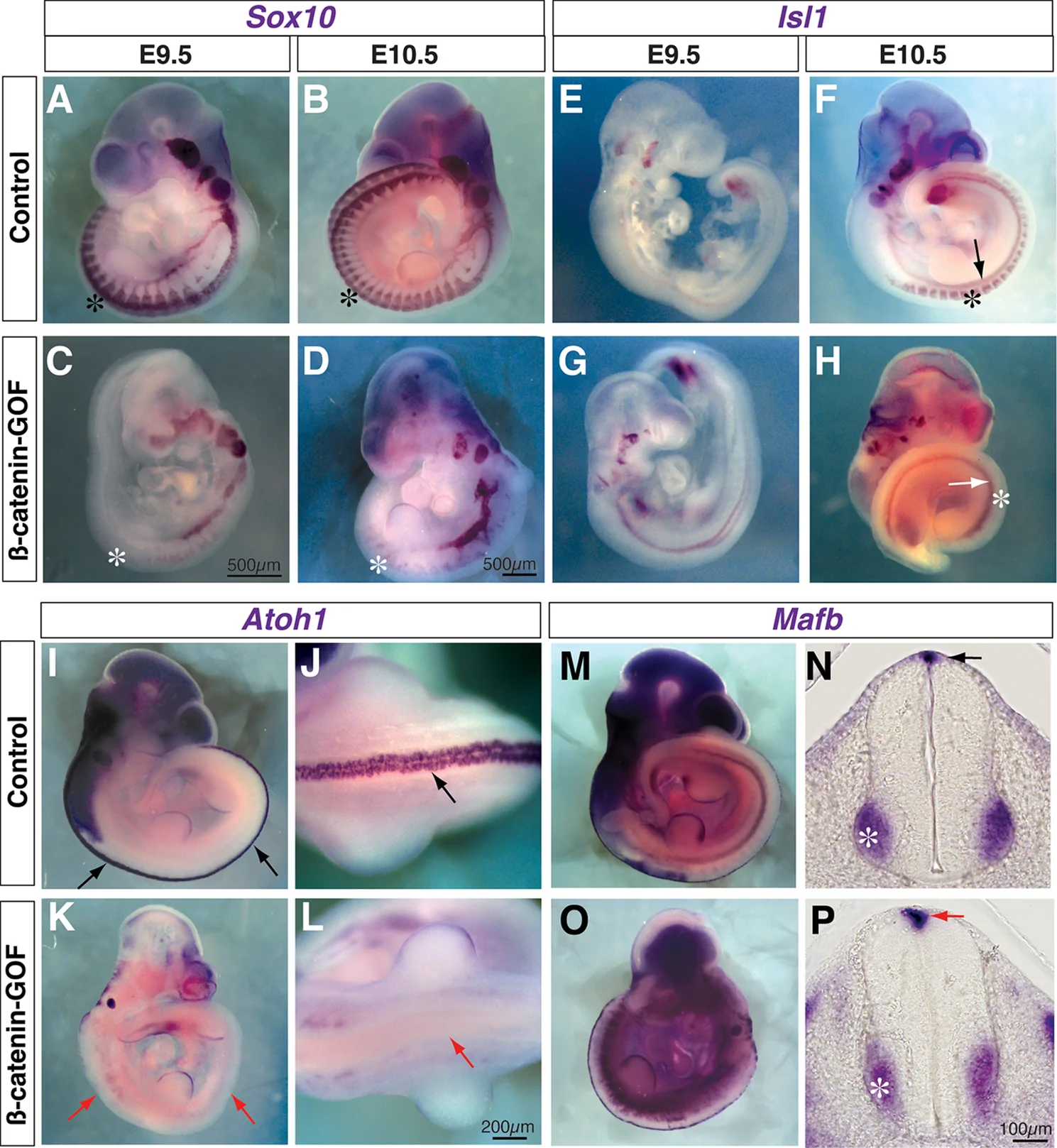
Defective neural crest cells and dorsal spinal interneuron progenitors in the ß-catenin-GOF mutants. **A**-**D **The expression of the neural crest marker gene *Sox10* is dramatically diminished in the cranial ganglia and abolished in the dorsal root ganglia (asterisks in **C**, **D**) of the mutants at E9.5 and E10.5. **E**-**H **Altered *Isl1* expression in the mutant embryos. *Isl1* expression is significantly diminished in the cranial ganglia and absent in the dorsal root ganglia (asterisks in **F**, **H**), but it is relatively conserved in the motor neurons of the ventral spinal cord in the E10.5 mutant embryos (arrows in **F**, **H**). **I**-**L** Loss of *Atoh1* expression in the dorsal spinal interneuron progenitors of the mutant embryos at E9.5 (arrows in **K**, **L**). **M**-**P** *Mafb* expression patterns remain relatively unchanged in the roof plate (arrows in **N**, **P**) and the ventral spinal interneurons and motor neurons (asterisks in **N**, **P**) of the ß-catenin-GOF mutants at E9.5. **A**-**I**, **K**, **M**, **O**, sagittal views; **J**, **L**, dorsal spinal views; **N**, **P**, transverse spinal sections

We have previously demonstrated that the Msh homeobox gene *Msx1* is transcriptionally activated by canonical Wnt/ß-catenin signaling during craniofacial development [27] and is significantly downregulated in the dorsal neural tube including roof plate in the ß-catenin-LOF mutants [22]. At E9.5, *Msx1* is significantly upregulated, visibaly in small clusters of migratory neural crest cells at thoracic neural tube level of the ß-catenin-GOF mutants (Fig. 2E, I). At E10.5, *Msx1* is also significantly upregulated, notably in all migratory neural crest cells in the entire spine region of the mutants (Fig. 2F, J). The *Msx1*-expressing domain in the dorsal neural tube of the mutants is much wider than in the control embryos and *Msx1* expression is dramatically upregulated in the migratory neural crest cells and dorsal root ganglia (DRG) in the ß-catenin-GOF at E10.5 (Fig. 2G, H). It has been shown that Msx1 acts upstream Pax3 to induce neural crest formation [34]. *Msx1* is not normally expressed in the migratory neural crest cells and DRG of the control embryos (Fig. 2A-D). The unexpected *Msx1* expression in the migratory neural crest cells and DRG suggest a cell fate alteration or defective differentiation of neural crest cells in the ß-catenin-GOF mutants. A previous study examined a neural crest marker *Erbb3* expression in the *ß-catenin* and *Pax3* double mutants and suggested that ß-catenin gain of function exacerbates the effect of *Pax3* mutation on neural crest cell migration [35]. In the current study, we examined the Sry-box transcription factor Sox10 that plays critical roles in neural crest development and is a definitive marker for migratory neural crest cells and their derivatives [36, 37]. At E9.5 and E10.5, *Sox10* is expressed exclusively in sensory ganglia (Fig. 3A, B), but its expression is dramatically diminished in cranial ganglia and is nearly absent in the DRGs of the ß-catenin-GOF mutants (Fig. 3C, D). The LIM-homeodomain transcription factor *Isl1* is expressed in all peripheral neurons [38], and it is evident in cranial ganglia from E9,5 and in DRG from E10.5 of the control embryos (Fig. 3E, F). *Isl1* expression remains unchanged in the ventral spinal motoneuron progenitors of the mutants at E9.5 and E10.5 (Fig. 3G, H). However, it is significantly diminished in cranial ganglia and absent in DRG of E10.5 ß-catenin-GOF mutants (Fig. 3H). Together with the ectopic expression of the neural crest inducer *Msx1* in the migratory stream and DRGs, the abolished *Sox10* and *Isl1* expression in these regions suggest that neural crest cell differentiation is disrupted during or after migration and DRG formation in the mutants.

### Defective dorsal spinal interneuron patterning in ß-catenin stabilized mutants

The roof plate is a crucial organizer to regulate dorsal spinal interneuron identities [6]. The bHLH transcription factor Atoh1 (previously Math1) is specifically expressed in the dorsal interneuron progenitors flanking the roof plate [39]. *Atoh1* expression in the spinal interneuron progenitors is completely lost in the ß-catenin-GOF mutants (Fig. 3I-L). In contrast, the transcription factor *Mafb* is expressed in the ventral spinal interneurons and motoneurons [40], and is not altered in the ß-catenin-GOF mutants (Fig. 3M-P). Notably, *Mafb* is also a roof plate maker, which remains in the roof plate of the ß-catenin-GOF mutants compared to the littermate controls (Fig. 3N, P). However, RT-qPCR results show reduced mRNA levels of both *Atoh1* and *Mafb* in the mutants at both ages (Supplementary Fig. S1). These results suggest that the dorsal interneuron patterning is disrupted by ß-catenin stabilization in the Pax3-expressing dorsal neural tube.

### Uniquely altered roof plate gene expression patterns in ß-catenin stabilized mutants

**Fig. 4:**
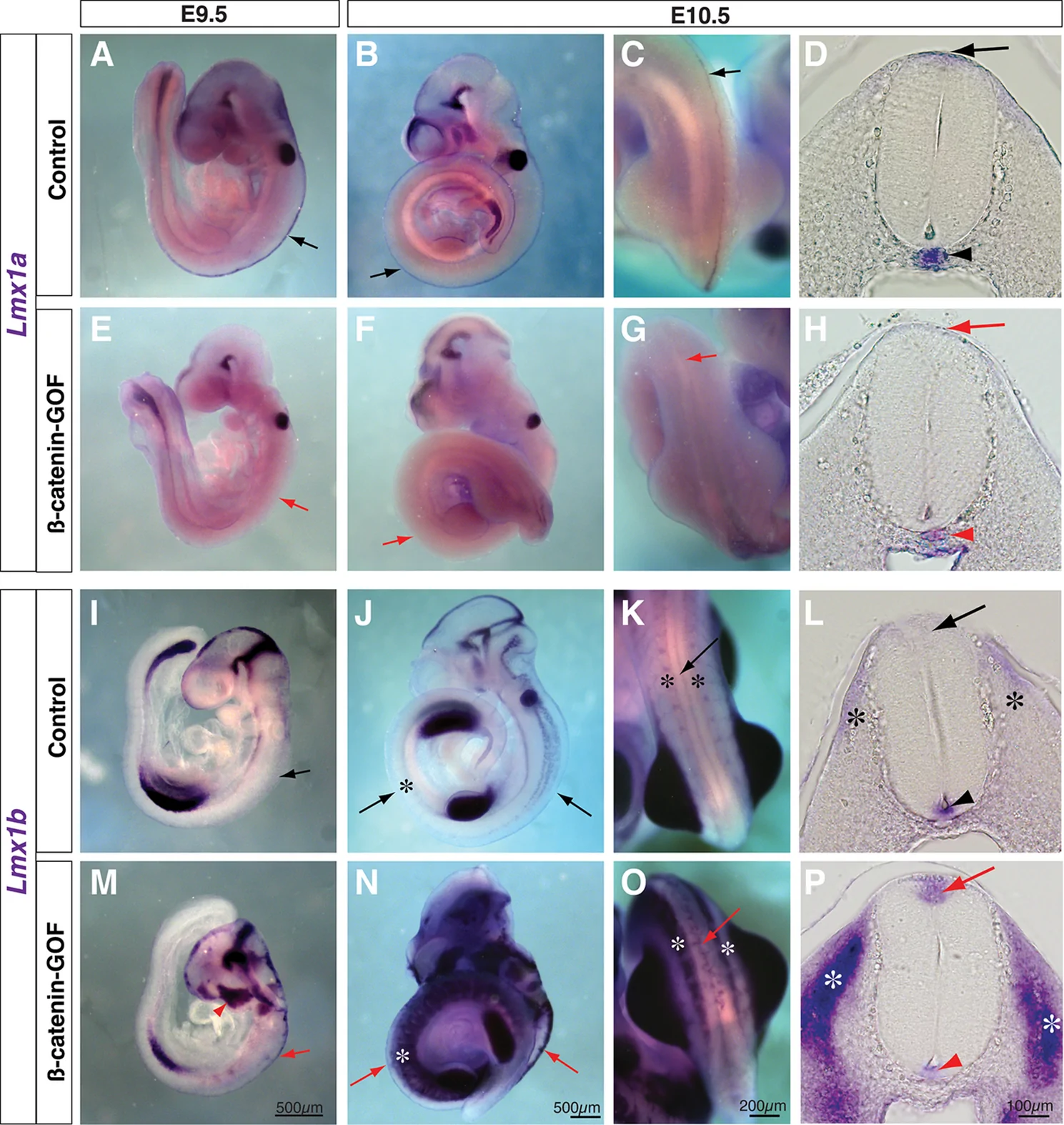
Cell fate alterations of the roof plate and migratory neural crest in the ß-catenin-GOF mutants. **A**-**H** *Lmx1a* expression is lost in the roof plate (red arrows in **E**-**H**) of the E9.5 and E10.5 mutants. Note diminished but remained *Lmx1a* expression in the notochord (red arrowhead in **H**). **I**-**P** ectopic expression of *Lmx1b* in the roof plate and dorsal root ganglia of the E9.5 and E10.5 mutants. Note ectopic expression of *Lmx1b* in the roof plate (red arrows in **M**-**P**) and migratory neural crest cells (white asterisks in **N**-**P**), and diminished but remained expression of Lmx1b in the floor plate (red arrowhead in **P**) of the mutants. Left two columns, lateral views of whole mouse embryos; **C**, **G**, **K**, **O**, dorsal spine views of respective E10.5 embryos; **D**, **H**, **L**, **P**, transverse sections at thoracic spine level at E10.5

**Fig. 5:**
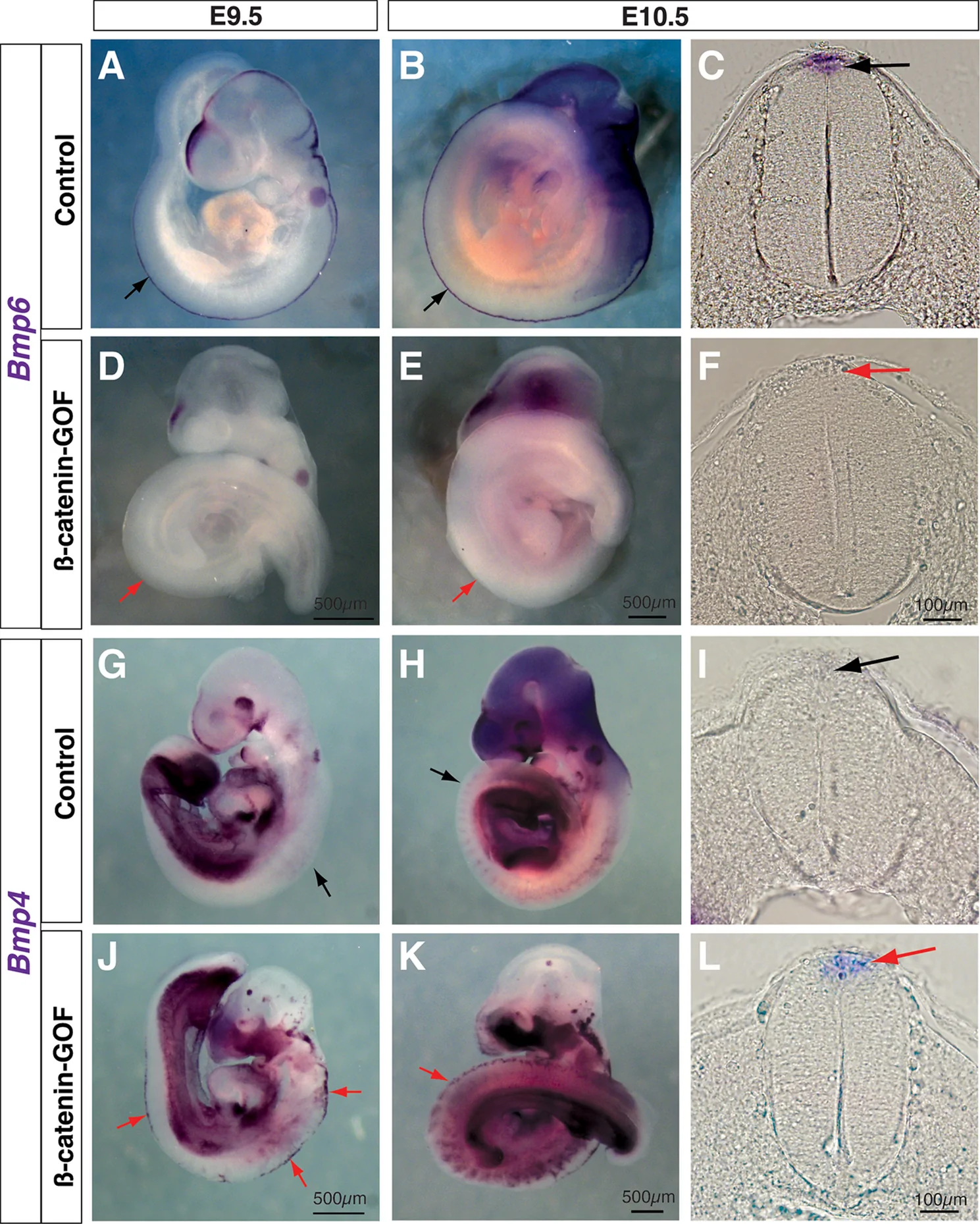
Swapped expression patterns of *Bmp4* and *Bmp6* in the roof plate of ß-catenin-GOF mutants. **A**-**F** Abolished expression of *Bmp6* (arrows in **D**-**F**) in the roof plate of the mutants at E9.5 and E10.5. **G**-**L** Ectopic *Bmp4* expression (arrows in **J**-**L**) in the roof plate of the mutants at E9.5 and E10.5. Left and middle columns, lateral views of whole embryos; **C**, **F**, **I**, **L**, transverse sections at thoracic spine level at E10.5

Although *Mafb* expression remains in the roof plate of the ß-catenin stabilized mutants, defective dorsal spinal interneuron patterning and neural crest cell differentiation suggest a root cause in the roof plate of the mutants. The LIM homeobox gene *Lmx1a* is required for roof plate formation and is a representative marker for mature roof plate [4, 11]. Lmx1a is expressed in the spinal roof plate of E9.5 and E10.5 control embryos (Fig. 4A-D) and its roof plate expression is lost in the ß-catenin-GOF mutants but remains in other regions including in the notochord (Fig. 4E-H). Its homologue *Lmx1b* is barely detectable in the spinal roof plate, but it is clearly expressed in the floor plate and mildly in the migratory neural crest cells of the control mouse embryos (Fig. 4I-L). Intriguingly, *Lmx1b* is dramatically upregulated or ectopically expressed in the mutant roof plate and DRG of the ß-catenin-GOF embryos (Fig. 4M-P). RT-qPCR results show no significant changes of *Lmx1a* at both ages and a statistically significant decrease of *Lmx1b* at E9.5 but not E10.5 in the mutant samples compared to that in the littermate controls (Supplementary Fig. S2). It has been shown that Bmp signaling can induce *Lmx1a* for roof plate formation [11]. *Bmp6* is restrictively expressed in the roof plate of E9.5 and E10.5 control embryos (Fig. 5A-C), which is absent in the entire roof plate (except a small region in the dorsal forebrain) along the anterior-posterior body axis in the ß-catenin-GOF mutants (Fig. 5D-F). However, *Bmp4* is ectopically expressed in the spinal roof plate of both E9.5 and E10.5 ß-catenin-GOF mutants (Fig. 5G-L). RT-qPCR results confirmed a decrease of *Bmp6* and an increase of *Bmp4* in the mutant embryos at E10.5 but not at E9.5 (Supplementary Fig. S3). The swapped expression patterns of *Bmp6* and *Bmp4* suggest a cell fate alteration of the roof plate in the ß-catenin stabilized mutants.

### Abolished expression of *Wnt1* and *Wnt3a* in ß-catenin stabilized roof plate

**Fig. 6:**
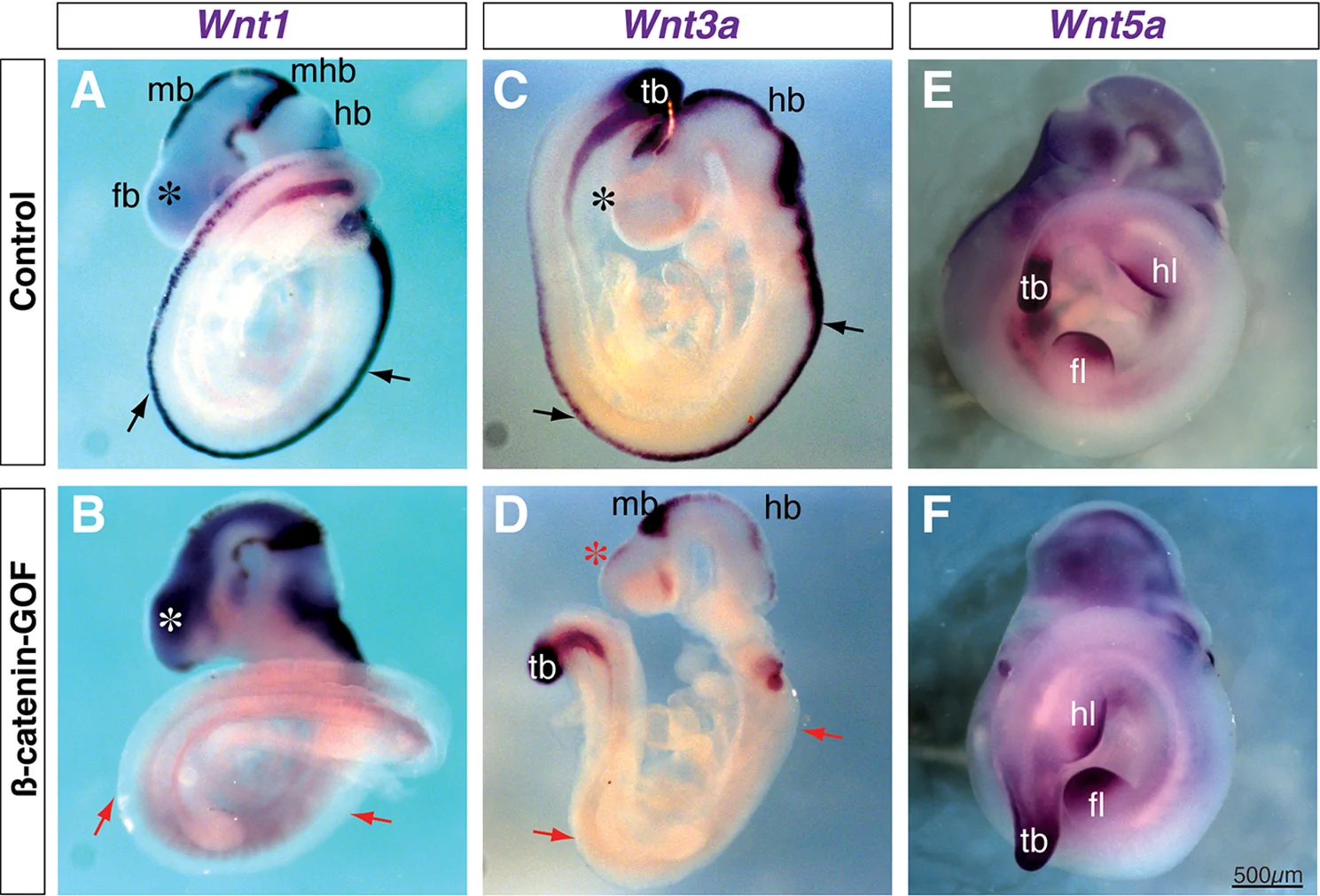
Abolished expression of *Wnt1* and *Wnt3a* but not *Wnt5a* in E9.5 ß-catenin-GOF embryos. **A**, **B** *Wnt1* expression is completely lost in the roof plate of the entire spinal regions in the mutants (arrows in **B**). Note the expanded *Wnt1* expression (asterisk in **B**) in the forebrain and facial regions. **C**, **D** *Wnt3a* expression is completely lost in the roof plate and dorsal neural tube of the entire spinal regions (arrows in **D**) and dramatically decreased in the hindbrain of the mutants. Note the relatively conserved *Wnt3a* expression in the tailbud and midbrain (**D**), and visible upregulation in the forebrain (asterisk in **D**). **E**, **F** Relatively unaltered *Wnt5a* expression patterns in the mutants. fb, forebrain; fl., forelimb; hb, hindbrain; hl, hindlimb; mb, midbrain; mhb, midbrain-hindbrain boundary; tb, tailbud

The primary function of the roof plate is to produce morphogenetic Wnt and Bmp signals required for dorsal neural tube patterning [41]. We have shown the switched *Bmp4* and *Bmp6* expression patterns in the roof plate of the ß-catenin-GOF mutants (Fig. 5). To investigate whether or how stabilized ß-catenin affects Wnt production at the roof plate, we examined Wnt1 and Wnt3a, two representative canonical Wnt ligands that act through ß-catenin signaling and play synergetic roles in neural crest production, somite patterning, and dorsal spinal cord patterning [5, 7, 15]. *Wnt1* and *Wnt3a* are exclusively expressed in the dorsal midline of the wild-type neural tube at E9.5 (Fig. 6A, C), and both are completely lost in the spinal roof plate or dorsal neural tube of ß-catenin-GOF mutants (Fig. 6B, D). Notably in the mutant forebrain region, *Wnt1* expression is ectopically expanded and *Wnt3a* is mildly upregulated (Fig. 6B, D). *Wnt3a* expression in the midbrain, hindbrain, and tailbud is decreased but remained in the mutants compared to that in the littermate controls (Fig. 6A-D). In contrast, the expression patterns of a noncanonical *Wnt5a* that is required for outgrowth of multiple embryonic structures [42] are relatively conserved in the mutant tail buds and limb buds at E9.5 (Fig. 6E, F). RT-qPCR results confirmed reductions of both *Wnt1* and *Wnt3a* at both ages and no changes of *Wnt5a* et E10.5 in the mutants (Supplementary Fig. S4). Together, these results suggest that constitutive activation of ß-catenin suppresses Wnt ligand productions in the spinal roof plate.

### Msx1-dependent and -independent modulations of the key regulatory genes abolished in the ß-catenin stabilized mutants

**Fig. 7:**
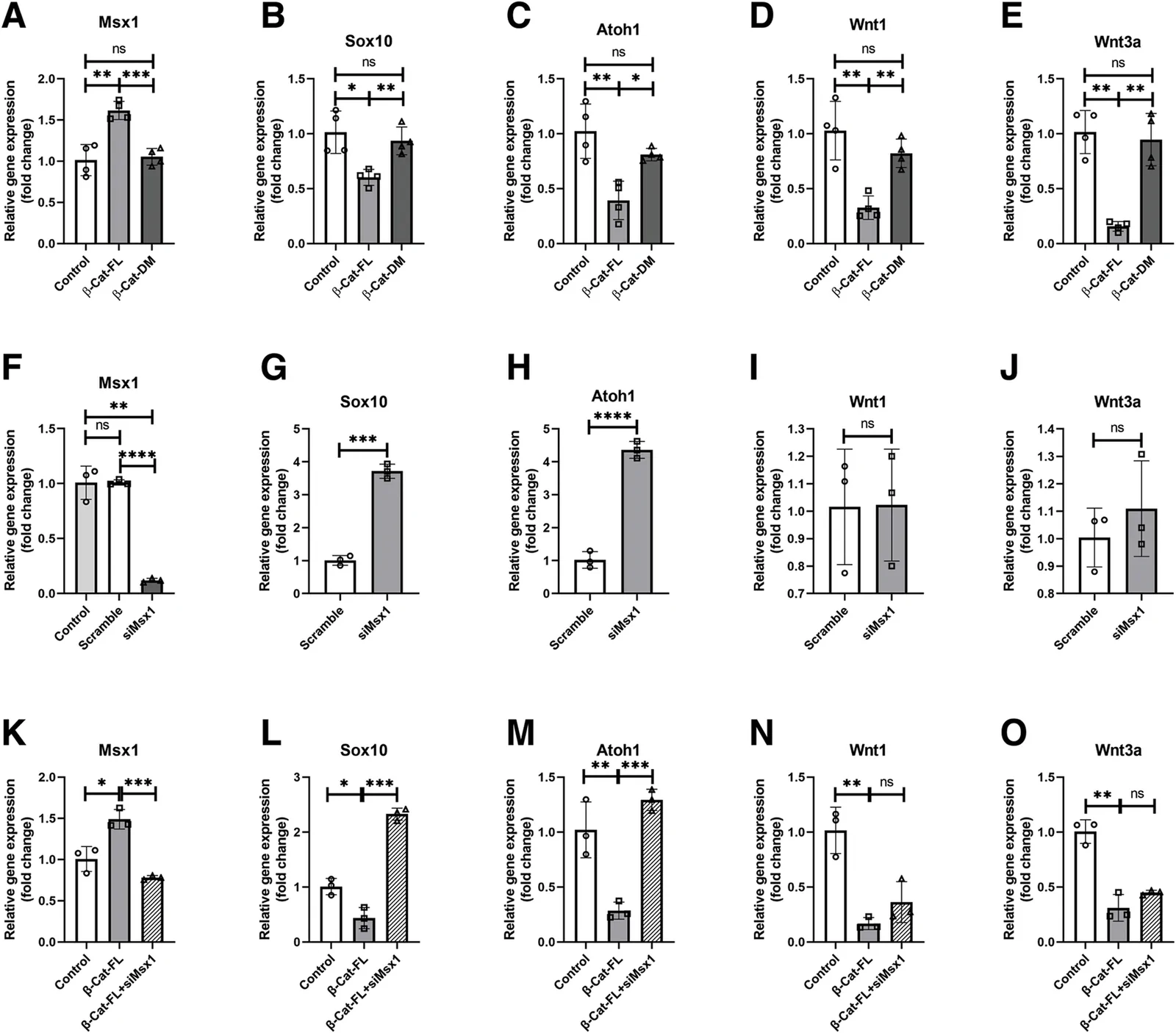
In vitro modulations of candidate downstream effectors by ß-catenin signaling and Msx1-knockdown in the neural crest O9-1 cell line. **A **RT-qPCR results demonstrate significant upregulation of *Msx1* mRNA level after transfection with the full-length “wild-type” *ß-catenin* (*ß-catenin-FL*), but not the signaling mutants of *ß-catenin-dm* constructs. **B**-**E** *Sox10*,* Atoh1*,* Wnt1*, and *Wnt3a* mRNA levels are downregulated by the full-length *ß-catenin-FL*, not by *ß-catenin-dm* signaling mutants. **F** Efficient knockdown of *Msx1* mRNAs by the short interfering RNA of *siMsx1* but not by the scrambled RNAi. **G**, **H** *Sox10* and *Atoh1* are significantly upregulated by *siMsx1*. **I**, **J** *Wnt1* and *Wnt3a* are not altered by *siMsx1*. **K** Concurrent transfection of *ß-catenin-FL* and *siMsx1* normalized *Msx1* expression level. **L**, **M** Diminished *Sox10* and *Atoh1* by *ß-catenin-FL* are significantly upregulated by concurrent *siMsx1*. **N**, **O** Diminished *Wnt1* and *Wnt3a* by *ß-catenin-FL* are not rescued by *siMsx1*. dm, double signaling mutants; FL, full length; ns, no statistical significance; *, *P* < 0.05; **, *P* < 0.01; ***, *P* < 0.001; ****, *P* < 0.0001

Based on the facts that *Msx1* is dramatically upregulated in the ß-catenin stabilized mutants (Fig. 2) and Msx1 is a known transcriptional repressor during embryonic development [43–46], we reasoned that the ectopically upregulated Msx1 represses a set of downstream target genes in the ß-catenin stabilized mutants. To test this hypothesis, we conducted a series of in vitro experiments using O9-1 mouse neural crest cells [28] to address the relationships among ß-catenin and Msx1 with 4 representative candidate target genes, including the neural crest differentiation factor *Sox10*, the dorsal spinal neuronal progenitor specifier *Atoh1*, and the roof plate-specific Wnt ligand genes *Wnt1* and *Wnt3a*, which are dramatically attenuated in the ß-catenin stabilized mutants. In agreement with our in vivo findings, transfection with the full-length cDNA of ß-catenin upregulates *Msx1* expression and represses *Sox10*, *Atoh1*, *Wnt1*, and *Wnt3a* in the O9-1 cells (Fig. 7A-E). In contrast, transfection with a signaling-specific mutant form of ß-catenin (ß-catenin-dm) [16] shows no effects on these genes (Fig. 7A-E). Alternatively, *Msx1* knockdown (*siMsx1*) causes dramatic upregulation of *Sox10* and *Atoh1*, but not *Wnt1* and *Wnt3a* in the O9-1 cells (Fig. 7F-J). Significantly, concurrent transfection of *siMsx1* and the full-length *ß-catenin* can normalize *Msx1* expression (Fig. 7K) and rescue or upregulate the diminished expression levels of *Sox10* and *Atoh1*, but not *Wnt1* and *Wnt3a* by ß-catenin alone in the O9-1 cells (Fig. 7L-O). Together, these results suggest that the attenuation of *Sox10* and *Atoh1*, not *Wnt1* and *Wnt3a*, in ß-catenin stabilized mutants is mediated by the repressive role of the upregulated Msx1. We conducted similar experiments on other crucial genes altered in the ß-catenin stabilized mutants and confirmed that ß-catenin signaling activates *Axin2*, *Cdx2*, *Pax3*, *Bmp4*, and possibly *Lmx1b*, but not *Isl1*, *Lmx1a*, or *Bmp6* in O9-1 cells (Supplementary Fig. S5A-H). Meanwhile, *Msx1* knockdown also diminishes the known Wnt/ß-catenin signaling target genes, *Axin2*, *Cdx2*, *Pax3* and *Bmp4* in vitro (Supplementary Fig. S5I-P), which may warrant further investigations.

### *Sox10* and *Atoh1* are transcriptionally repressed by Msx1

**Fig. 8:**
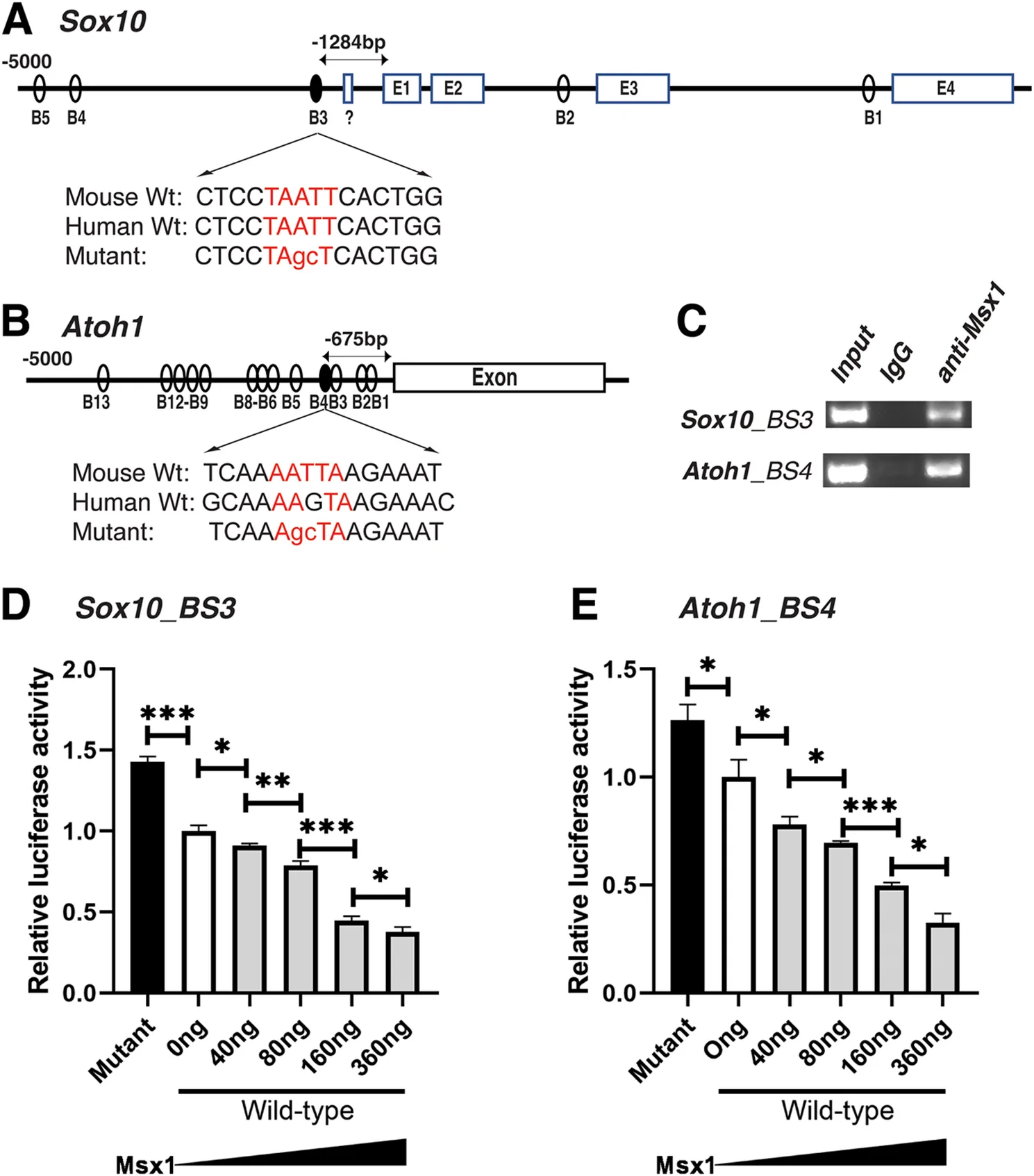
Transcriptional repression of *Sox10* and *Atoh1* by Msx1. **A **Multiple Msx1 binding sites are found, including B1 in intron 3, B2 in intron 2 and B3-B5 within the 5 kb upstream promoter region of *Sox10* gene. The B3 motif is conserved in the human *SOX10* gene. **B** 13 Msx1 binding sites are found within the 5 kb upstream promoter region of *Atoh1* gene. The B4 motif shows one base pair different with that in human *Atoh1* gene. **C** Chromatin immunoprecipitation demonstrates the specific recruitments of the binding site BS3 in *Sox10* and BS4 in *Atoh1* by Msx1 antibodies, but not the non-specific IgG. **D**, **E** Luciferase reporter assays demonstrate dose-dependent repression effects of Msx1 on the constructs driven by the wild-type *Sox10_BS3* and *Atoh1_BS4*. In contrast, the constructs with respectively mutated motifs demonstrate upregulation of the luciferase reporter activities. *, *P* < 0.05; **, *P* < 0.01; ***, *P* < 0.001

To address how *Sox10* and *Atoh1* are repressed by Msx1, we searched presumptive promoter regions up to 5,000 bp 5’ upstream of these genes and found 5 candidate Msx1 binding sites (*BS1-5* or *B1-5*) with a consensus sequence of *AATTA* [44] in *Sox10* and 13 candidate Msx1 binding sites (*BS1-13*) in the *Atoh1* region (Fig. 8A, B). Chromatin immunoprecipitation (ChIP) experiments revealed that Msx1 antibody can specifically pull down *BS3* in *Sox10* and *BS4* in *Atoh1* presumptive promoter regions (Fig. 8C). We found that these Msx1 binding sites are conserved in their respective human homologs (Fig. 8A, B; Supplementary Figs. S6 & S7). We then created luciferase reporter constructs containing the two binding sites validated by ChIP and found that Msx1 can dose-dependently repress the reporter activities of *Sox10_BS3* and *Atoh1_BS4* constructs. Conversely, the constructs with mutated binding sites upregulate reporter activities (Fig. 8D, E). These results strongly suggest that *Sox10* and *Atoh1* are direct repression targets of Msx1. We also conducted similar experiments on *Wnt1* and *Wnt3a* genes and found that although Msx1 antibody can recruit *BS5* in *Wnt1* and *BS1* in *Wnt3a* presumptive promoter regions, these binding sites are not conserved in respective human *Wnt1*/*Wnt3a* genes (Supplementary Fig. S8A-C). Moreover, Msx1 shows no or opposite effects on *Wnt1* and *Wnt3a* luciferase reporter activities in O9-1 cells (Supplementary Fig. S8D, E).

### *Wnt1* and *Wnt3a* are transcriptionally repressed by ß-catenin signaling

**Fig. 9:**
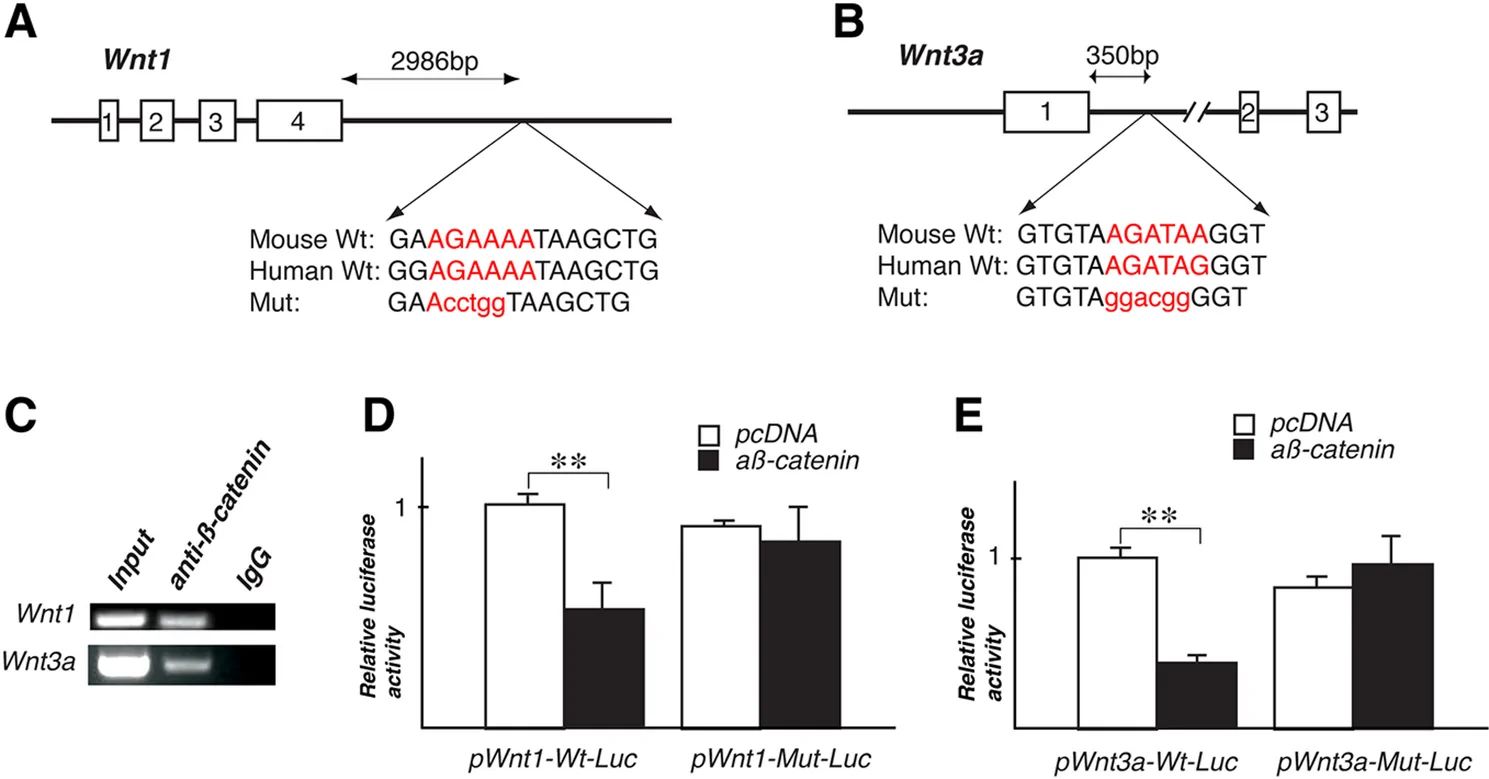
Transcriptional repression of *Wnt1* and *Wnt3a* by ß-catenin. **A** A repression motif *AGAAAA* is found in the downstream region of mouse *Wnt1* gene, 2986 bp from the end of exon 4, which is conserved in the human *WNT1* gene. **B** A slightly different repression motif *AGATAA* is found in mouse *Wnt3a* intron 1 with 350 bp distance from the end of exon 1. This motif shows one base pair different with that in human *WNT3A* gene. 1–4, exon 1–4; Wt, wild-type motif; Mut, mutated motif. **C** Chromatin immunoprecipitation demonstrates the specific recruitments of the repression motifs by ß-catenin antibodies, but not the non-specific IgG, from the wild-type spinal regions of E9.5 mouse embryos. **D**, **E** Luciferase reporter assays demonstrate diminished activation of the constructs with the wild-type, but not the mutated motifs after co-transfection of the *active ß-catenin* (*aß-catenin*) cDNAs. The *pcDNA* vector was used as the control. **, *P* < 0.01

**Fig. 10:**
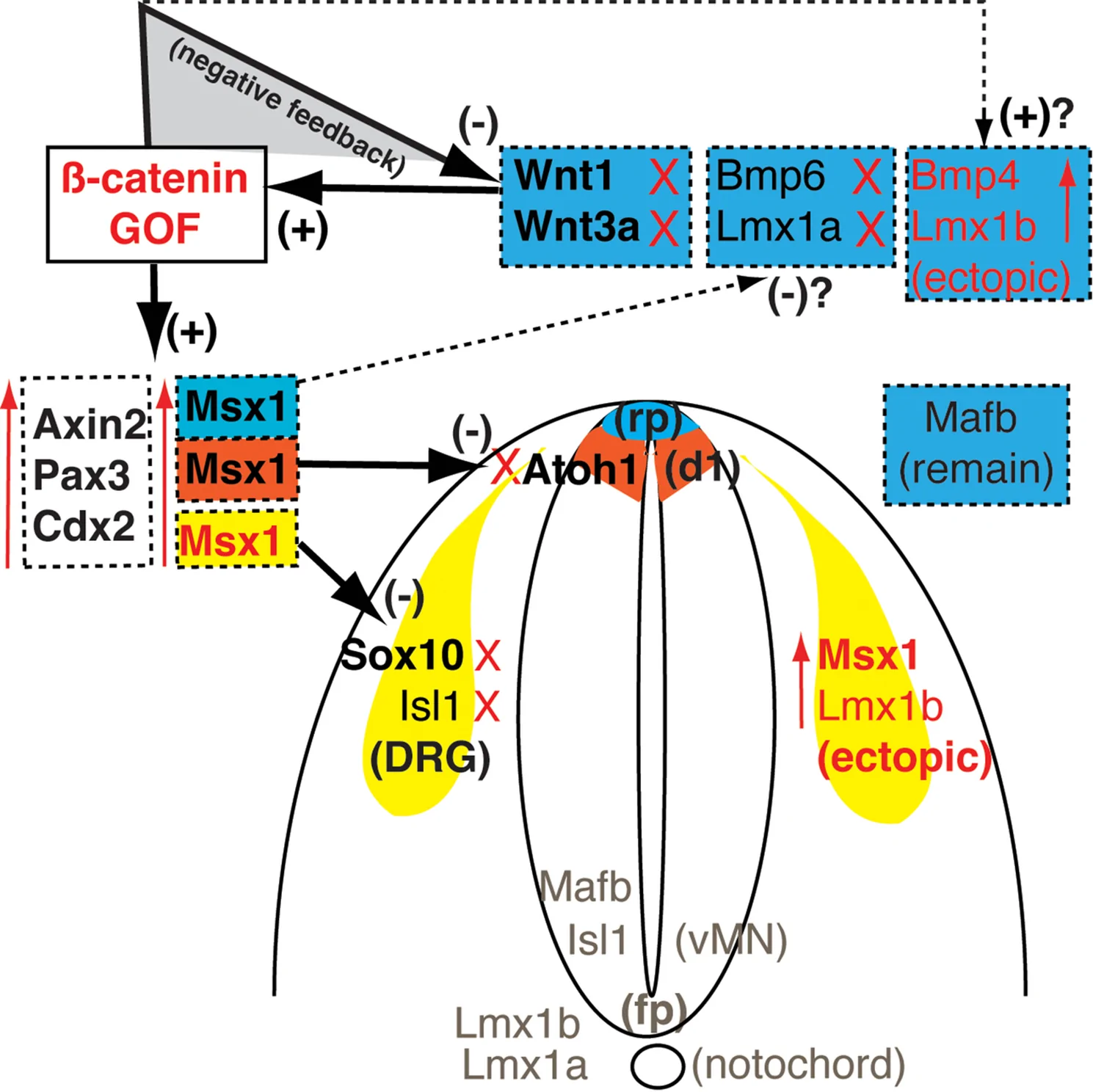
Summary and mechanistic illustration of the current study. Constitutively activated ß-catenin in the dorsal neural tube causes excessive expression of *Msx1* that subsequently represses *Atoh1* in the dorsal neuronal progenitors and represses *Sox10* in the neural crest cells during migration and differentiation in the dorsal root ganglia. Meanwhile, stabilized ß-catenin represses *Wnt1* and *Wnt3a* in the roof plate through transcriptional repression of the ß-catenin/Tcf complex, which forms a novel negative feedback loop of the canonical Wnt/ß-catenin signaling pathway. Stabilized ß-catenin also causes swapped expression patterns of *Bmp6* (abolished) with *Bmp4* (ectopic), and *Lmx1a* (abolished) with *Lmx1b* (ectopic) in the roof plate through a possible mechanism of concurrent repression by Msx1 (on Bmp6 and Lmx1a) and upregulation by ß-catenin (on *Bmp4* and *Lmx1b*). Several other known downstream targets of Wnt/ß-catenin signaling, including *Axin2*, *Pax3*, and *Cdx2* are upregulated in the dorsal neural tube, and *Cdx2* is ectopically expressed in the anterior spinal neural tube of the ß-catenin stabilized mutants. Several gene expression patterns in the ventral neuronal domains and the floor plate are not affected. Solid black arrows indicate the experimentally demonstrated regulations and dashed arrows indicate unconfirmed regulations. Red arrows indicate upregulation or ectopic expression. X, abolished expression; (+), positive regulation; (-), negative regulation; d1, dorsal interneuron domain 1; DRG, dorsal root ganglia; fp, floor plate; GOF, gain-of-function; rp, roof plate; vMN, ventral motor neuron domain

Canonical Wnt/ß-catenin signaling is well known for transcriptional activation of downstream target genes by recruiting ß-catenin and transcription factor complex to the Tcf/Lef1-binding sites or Wnt responsive elements (WREs) with a consensus of CTTGWWS (W = A/T, S = C/G) in the promoter region [47]. A different WRE with a consensus of AGAWAW shows repression of target genes when Wnt/ß-catenin signaling is activated in the fly [48]. To reveal the molecular mechanism underlying Wnt ligand repression by active ß-catenin in the roof plate, we searched the repression motif in non-coding regions of *Wnt1* and *Wnt3a* genes and found *AGAAAA* in *Wnt1* downstream and *AGATAA* in *Wnt3a* intron 1, which are conserved in respective human genes (Fig. 9A, B). Chromatin immunoprecipitation assays demonstrate specific bindings of the ß-catenin/Tcf complex to these repression motifs in tissue samples isolated from the spinal region of E9.5 mouse embryos (Fig. 9C). Luciferase reporter assays demonstrate diminished activation of the construct with the wild-type, but not the mutated repression motifs after co-transfection of the active *ß-catenin* cDNAs (Fig. 9D, E). These results suggest that *Wnt1* and *Wnt3a* are direct downstream target genes of transcriptional repression by ß-catenin signaling, revealing a novel negative feedback loop of the canonical Wnt/ß-catenin signaling pathway at the Wnt ligand level (Fig. 10).

## Discussion

### Constitutive activation of ß-catenin alters roof plate marker gene expression patterns

The current study detected uniquely altered expression patterns of representative roof plate markers and regulatory genes in the ß-catenin stabilized mutants, which include swapped expression patterns of roof plate marker genes *Bmp6* and *Lmx1a* with their homologues *Bmp4* and *Lmx1b*, and ectopic expression of a caudal-type homeobox gene *Cdx2* in more anterior regions of spinal roof plate. The LIM-homeobox transcription factors Lmx1a and Lmx1b have complementary expression patterns and functions during CNS development [49]. Lmx1a is predominantly expressed in the spinal neural tube and it is required for spinal roof plate formation [12], while Lmx1b is exclusively expressed in the anterior roof plate and the mid-hindbrain boundary region and plays a synergetic role with Lmx1a in hindbrain roof plate formation and cerebellar growth [14]. In this study we found that *Lmx1a* expression is completely lost but *Lmx1b* is ectopically expressed in the spinal roof plate of the ß-catenin stabilized mutants, which may indicate a functional compensation of these Lmx1 homologues. However, we previously found that the expression patterns of *Lmx1a* and *Lmx1b* are not altered in the roof plate of the *Pax3-Cre;ß-catenin-LOF* mutants [22]. These results suggest a role of proper ß-catenin signaling in maintaining roof plate cell fate, rather than transcriptional activation of roof plate marker genes as downstream targets. The cell fate alteration is further supported by the swapped expression patterns of *Bmp6* with *Bmp4* in the spinal roof plate of the ß-catenin stabilized mutants. It is unclear why the mutant roof palate is still able to produce Bmps but differentially regulates the different family members. Unlike in the mouse neural tube, Bmp4 is predominantly expressed in the chicken roof plate [50], which may provide a clue for of the susceptibility of *Bmp4* to be activated in the mouse roof plate of the ß-catenin stabilized mutants. Intriguingly, the caudal-type homeobox gene *Cdx2* is ectopically expressed in the anterior region of the spinal roof plate in the ß-catenin stabilized mutants, which can be partially explained by that *Cdx2* is a positive downstream target gene of Wnt/ß-catenin signaling in the dorsal posterior neuropore as demonstrated in our previous study [22], and constitutive activation of ß-catenin may maintain *Cdx2* expression in the anterior region after the neural tube has elongated.

### Constitutive activation of ß-catenin may not prevent neural crest formation and migration but alter cell fate specification and differentiation in the dorsal root ganglia and dorsal spinal cord

The current study demonstrates upregulation of *Pax3* and ectopic expression of *Msx1* in the migratory neural crest cells and dorsal root ganglia (DRGs) of the ß-catenin stabilized mutants. The upregulated expression of *Pax3* and *Msx1* caused by constitutive activation of ß-catenin is in line with our earlier findings that both *Pax3* and *Msx1* are positive downstream target genes of Wnt/ß-catenin signaling during neural tube closure and orofacial development [22, 27]. Both Pax3 and Msx1 are required for neural crest cell induction, and they are actively expressed in the pre-migratory neural crest cells, with Pax3 acting downstream of Msx1 as demonstrated in Xenopus [34]. However, *Msx1* is not normally expressed in the migratory neural crest cells and DRGs. Moreover, accompanying its ectopic expression in the mutant roof plate, *Lmx1b* is dramatically upregulated in migratory neural crest cells and DRGs of the mutants. The ectopic expression of *Msx1* and dramatic upregulation of *Lmxb1* in these regions suggest that constitutively stabilized ß-catenin promotes neural crest cell production and migration at the undifferentiated status into the DRGs (Fig. 10).

Indeed, we found that *Sox10* and *Isl1* are dramatically diminished in the neural crest-derived DRGs of the ß-catenin stabilized mutants. Sox10 is a definitive molecular marker for neural crest cells after dissociation from the neural tube and directly modulates transcription of *Erbb3* to regulate NCC development [35, 51, 52]. Sox10 is essential for neural crest development in many ways, including multipotency maintenance of neural crest stem cells, cell fate specification and differentiation of neural crest derivatives [37, 53–59]. Particularly, Sox10 is required for the development of the vagal and trunk neural crest derivatives, including enteric ganglia, pigment cells, and DRGs [36, 60]. In the developing DRGs, Sox10 plays a key role in specification of neural crest-derived sensory neurons [61]. Thus, missing Sox10 suggests defective specification of sensory neurons in the mutant DRGs. Supportively, we found that *Isl1* expression is missing specifically in the mutant DRGs, but not in the motoneurons of the ventral spinal cord. Isl1 plays a key role in differentiation of sensory neurons and early sympathetic neurons [38, 62]. Taken together, our results suggest that ß-catenin stabilization in the roof plate and neural crest cell lineage may not prevent or even may promote production of neural crest lineage cells that migrate out the dorsal neural tube to form the DRGs, but these *Msx1*-expressing neural crest lineage cells in the migratory stream and DRGs maintain an undifferentiated status like the pre-migratory neural crest precursors, which prevents sensory neuronal specification and differentiation in the mutants, evident by abolished expression of *Sox10* and *Isl1* in DRGs (Fig. 10). However, it requires further studies to reveal the impacts of the constitutively stabilized ß-catenin on neural crest production and migration.

In parallel with neural crest induction, the roof plate regulates dorsal spinal interneuron identities [6]. The current study selectively examined an exemplary marker gene *Atoh1* that is required for the specification of the dorsal interneuron progenitor d1 domains flanking the roof plate [39, 63]. Our results demonstrate that constitutively stabilized ß-catenin abolished *Atoh1* expression in the dorsal neural tube at E9.5. Following spinal cord development, d1 precursors will migrate ventrally into the intermediate gray to generate several subpopulations of interneurons in the spinocerebellar tracts for functional proprioception [64]. Interestingly, Atoh1 knockout did not prevent d1 progenitor generation, but it might disrupt d1 precursor migration and differentiation [64], which would be expected to occur in the ß-catenin stabilized mutants.

### Msx1-mediated repression of stabilized ß-catenin on specific transcription factors for neural crest cell and dorsal interneuron specification and differentiation

Our in vivo and in vitro results strongly suggest that the repression role of stabilized ß-catenin on *Sox10* in the DRG and *Atoh1* in the dorsal spinal progenitors is mediated by ectopically expressed Msx1. Conversely, *Sox10* expression remained in the cranial neural crest cells, but its migratory strips are disorganized in the double knockouts of *Msx1* and *Msx2* mutant mouse embryos [65]. Notably, Msx1 can bind to a presumptive enhancer region to promote *Atoh1* activities in vitro and that *Atoh1* expression is lost in the dorsal spinal cord of the *Msx1/Msx2* double knockout mouse embryos [66], suggesting that Msx1 can transcriptionally activate *Atoh1* expression. However, our in vivo results clearly demonstrate that regardless of the dramatic upregulation of *Msx1* expression, *Atoh1* is lost in the dorsal spinal progenitor domain of the ß-catenin-stabilized mutants. Our chromatin immunoprecipitation and promoter luciferase assays demonstrate that Msx1 can bind to the presumptive promoter regions of *Atoh1* and *Sox10*, and that Msx1 dose-dependently represses *Atoh1* and *Sox10* promoter activities. Our in vitro experiments further demonstrate that Msx1 knockdown significantly upregulates *Atoh1* and *Sox10* expression. The repression effects of *Atoh1* and *Sox10* by the full-length ß-catenin are diminished by ß-catenin-dm signaling mutants or rescued by concurrent Msx1 knockdown. The seemingly conflict results between ours and Duval et al. [66] suggest that the endogenous Msx1/Msx2 transcription factors may normally initiate *Atoh1* (but not *Sox10*) expression by binding to a 3’ *Atoh1* enhancer, but excess Msx1 due to ectopic activation then results in binding to the repression domain in the promoter region. A technical limitation of our in vitro experiments is the use of O9-1 cells that is ideal for neural crest cell studies but may not perfectly suited for roof plate or interneurons. Meanwhile, these in vitro findings need to be further validated by in vivo approaches, such as transgenic activation of Msx1 alone or conditional ablation of Msx1 in the ß-catenin stabilized mutant mouse embryos.

### A novel negative feedback loop of ß-catenin signaling by repressing Wnt ligand production

The current study strongly suggests that the dramatically attenuated Wnt1/Wnt3a expression in the ß-catenin-stabilized roof plate is a result of direct repression by the ß-catenin-mediated transcriptional regulation. A key function of the canonical Wnt/ß-catenin signaling pathway is to activate a wide range of downstream target genes through stabilized ß-catenin that enters into the nuclei and serves as a transcriptional co-activator by binding to the Tcf/Lef transcription factors [67]. Among the long list of the Wnt target genes (refer to Wnt homepage by Dr. Nusse at Standford University), several of them are Wnt pathway components, such as *Axin2* that encodes a crucial component of the ß-catenin destruction complex and can be directly activated by stabilized ß-catenin, forming a well-known negative feedback loop in the signaling pathway [33, 68, 69]. Other than as a transcriptional co-activator, ß-catenin can also act as a transcriptional repressor as clearly demonstrated in Drosophila [70]. Additional studies also suggest a repressive role of the ß-catenin-associated transcriptional complex [71]. For the first time, the current study demonstrates a role of the ß-catenin transcriptional complex on repressing Wnt1/Wnt3a expression, revealing a novel negative feedback loop of the Wnt/ß-catenin signaling pathway by modulating Wnt ligand production (Fig. 10), which may be applicable to other pathogenic conditions, such as cancer.

## Conclusions

This study revealed cell fate alterations in the dorsal neural tube derivatives caused by constitutively stabilized ß-catenin, which may act through direct repression of the ß-catenin transcriptional complex on Wnt1/Wnt3a production in the roof plate and indirect repression by ectopically upregulated Msx1 on neural crest cell differentiation in the migratory stream and dorsal root ganglia and on interneuron specification in the dorsal spinal precursors (Fig. 10). These results suggest that a finely tuned ß-catenin signaling level is crucial for proper cell fate determination and functional regulation of the roof plate precursor and its adjacent domains during vertebrate neural tube development.

## Supplementary Information

Supplementary Material 1.

## Data Availability

The raw data generated for this study are available from the corresponding author on reasonable request due to the large number and size of the files. The mouse lines need approval of the original distributors or can be acquired through the Jackson Laboratory. Other research materials including in situ probes, qPCR primers, cDNAs, siRNAs, and cell lines can be acquired through either major venders or original distributors, and can be partially shared by us when we have spare materials left.
