# Supplementary material for: Dual repression modes triggered by constitutively stabilized beta-catenin in the dorsal neural tube underlie cell fate alterations of the roof plate, neural crest, and dorsal interneurons
Source: Cell Biosci. 2026 Jun 30;16:107. doi: 10.1186/s13578-026-01615-x (PMC13584487; doi:10.1186/s13578-026-01615-x)
Supplement: Supplementary file 1 [file 13578_2026_1615_MOESM1_ESM.pdf]

Supplementary Fig. S1. RT-qPCR results of *Sox10*, *Isl1*, *Atoh1* and *Mafb* in the littermate controls and *ß-catenin-GOF* mutant embryos at E9.5 (A) and E10.5. *, $P < 0.05$; **, $P < 0.01$; ***, $P < 0.001$.

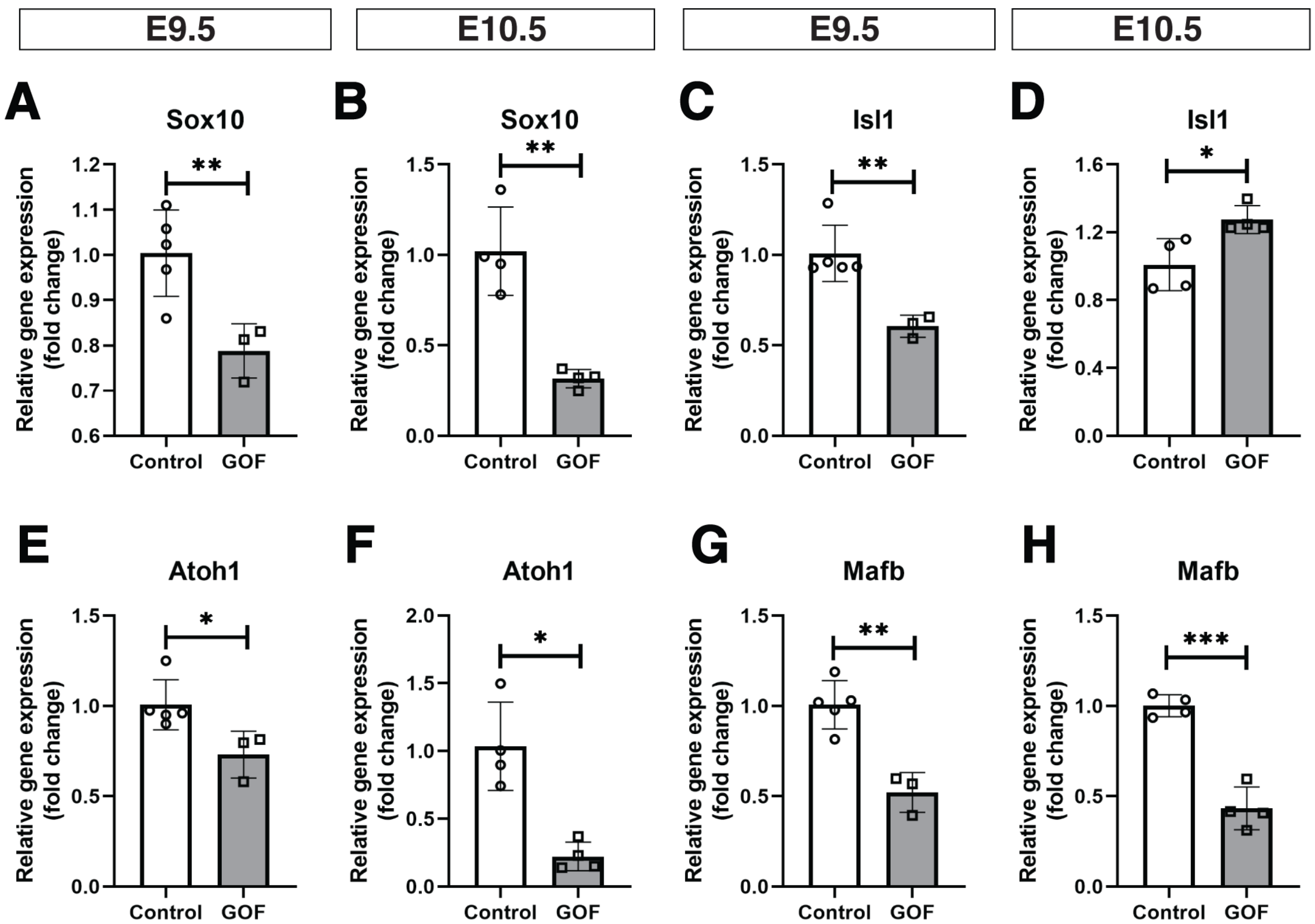

Supplementary Fig. S2. RT-qPCR results of *Lmx1a* and *Lmx1b*. (A,B) *Lmx1a* shows no significant changes (ns) in *ß-catenin-GOF* mutants at both E9.5 and E10.5. (C,D) *Lmx1b* in *ß-catenin-GOF* mutants is downregulated at E9.5 but no significant changes at E10.5. **, $P < 0.01$.

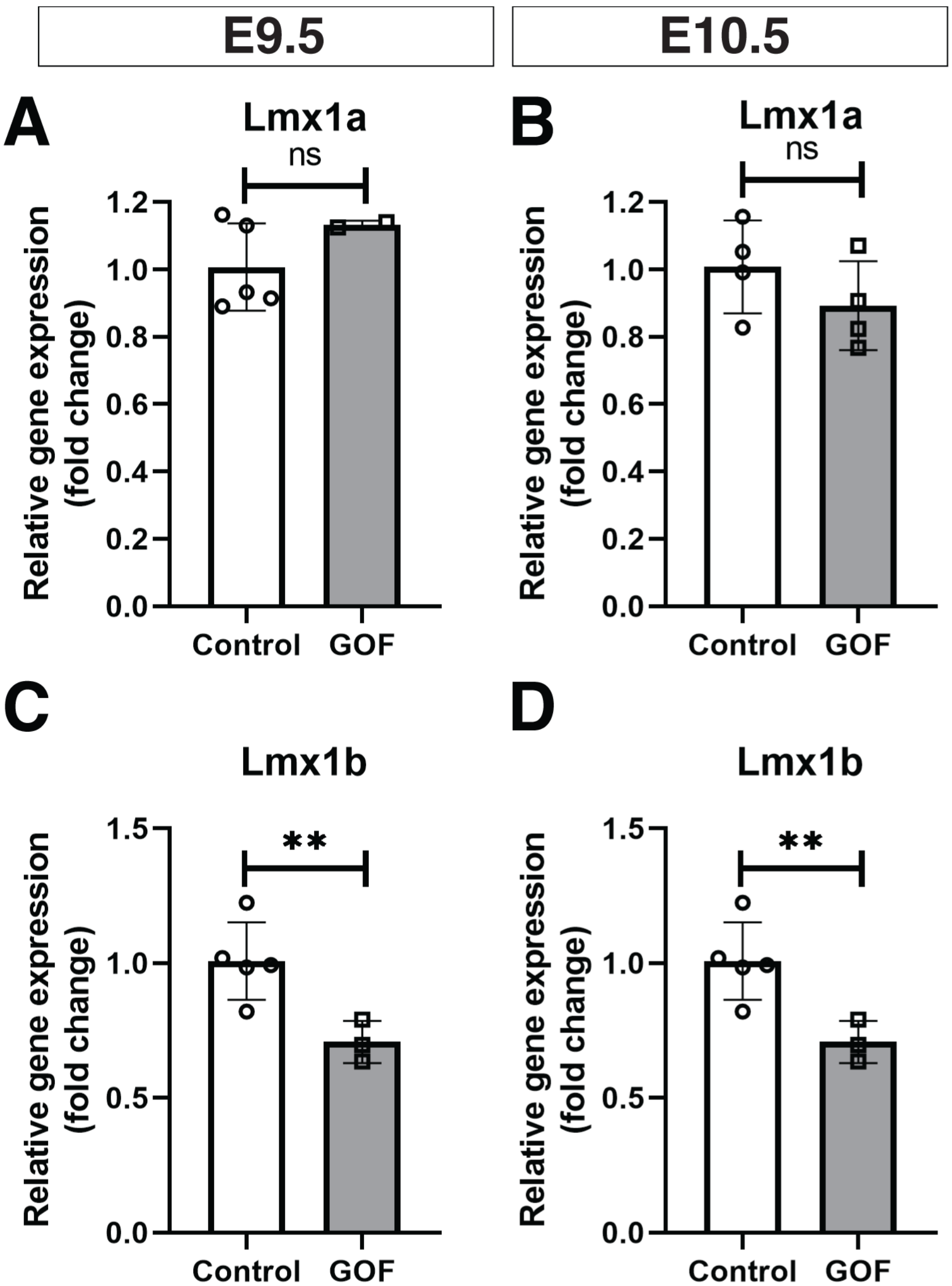

Supplementary Fig. S3. RT-qPCR results of *Bmp6* and *Bmp4*. (A,B) *Bmp6* in *ß-catenin-GOF* mutants shows no significant changes (ns) at E9.5 (A) while it is downregulated at E10.5 (B). (C,D) *Bmp4* is downregulated in *ß-catenin -GOF* mutants at E9.5 but it is upregulated at E10.5. *, $P < 0.05$; **, $P < 0.01$; ***, $P < 0.001$.

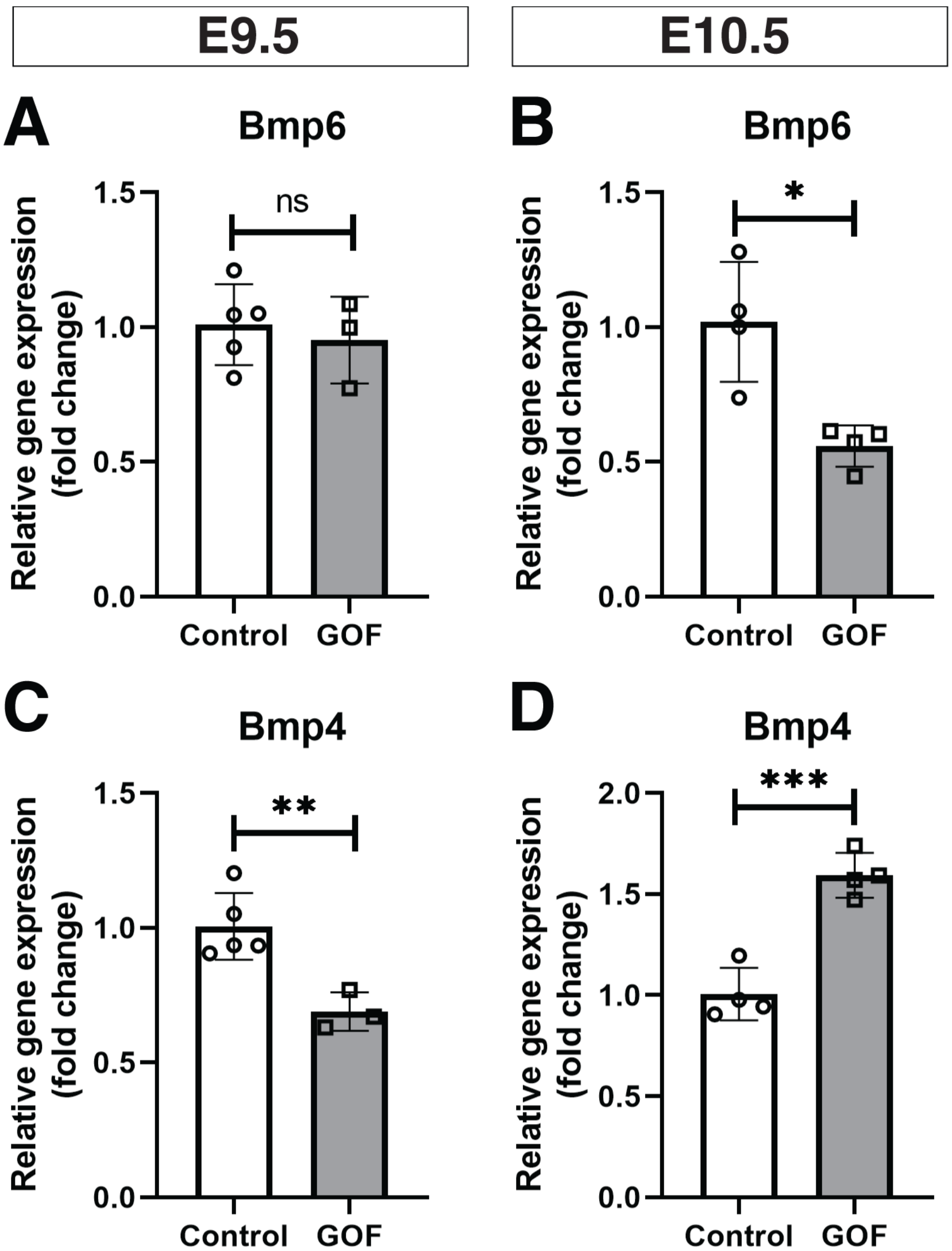

Supplementary Fig. S4. RT-qPCR results of *Wnts*. (A-D) Both *Wnt1* and *Wnt3a* are significantly downregulated in *ß-catenin-GOF* mutants at E9.5 and E10.5. (E,F) *Wnt5a* is downregulated at E9.5 but no significant changes (ns) at E10.5 in *ß-catenin-GOF* mutants. **, $P < 0.01$; ***, $P < 0.001$; ****, $P < 0.0001$.

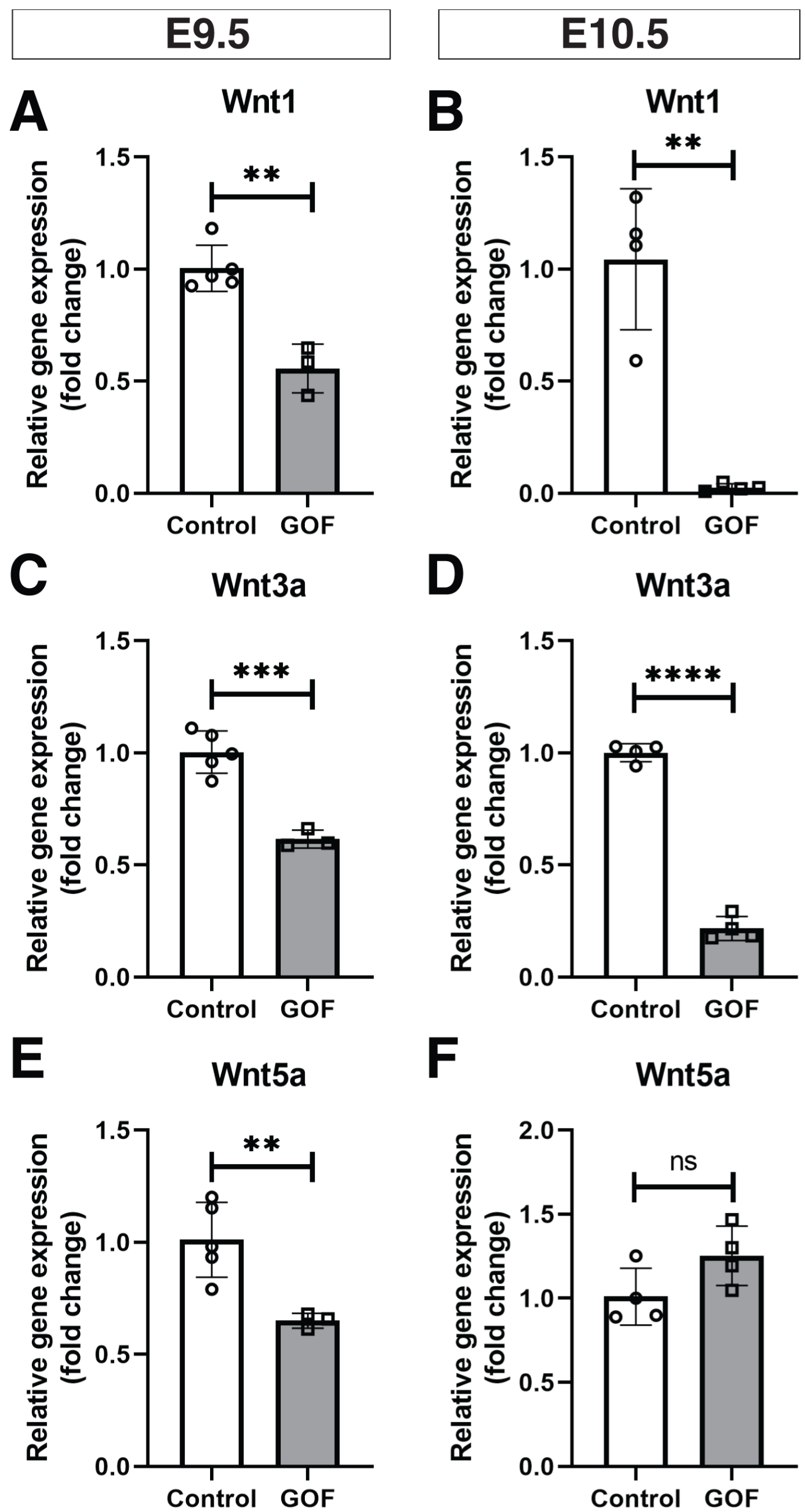

Supplementary Fig. S5. In vitro modulations of other significant genes by ß-catenin signaling and Msx1-knockdown in the neural crest O9-1 cell line. (A-H) RT-qPCR results show significant upregulations of *Axin2*, *Cdx2*, *Pax3*, and *Bmp4* by the full-length ß-catenin but no significant changes of these genes (except *Pax3* that is downregulated) by the signaling mutant ß-catenin-dm. In contrast, *Isl1*, *Lmx1a*, *Lmx1b*, and *Bmp6* are not significantly altered by either full-length ß-catenin or signaling mutants of ß-catenin-dm, except *Bmp6* that is downregulated by ß-catenin-dm**.** (I-P) siMsx1 knockdown also downregulates *Axin2*, *Cdx2*, *Pax3*, and *Bmp4*, and it may upregulate *Isl1*, *Lmx1a*, *Lmx1b*, and *Bmp6* without statistical significance. *, P < 0.05; **, P < 0.01.

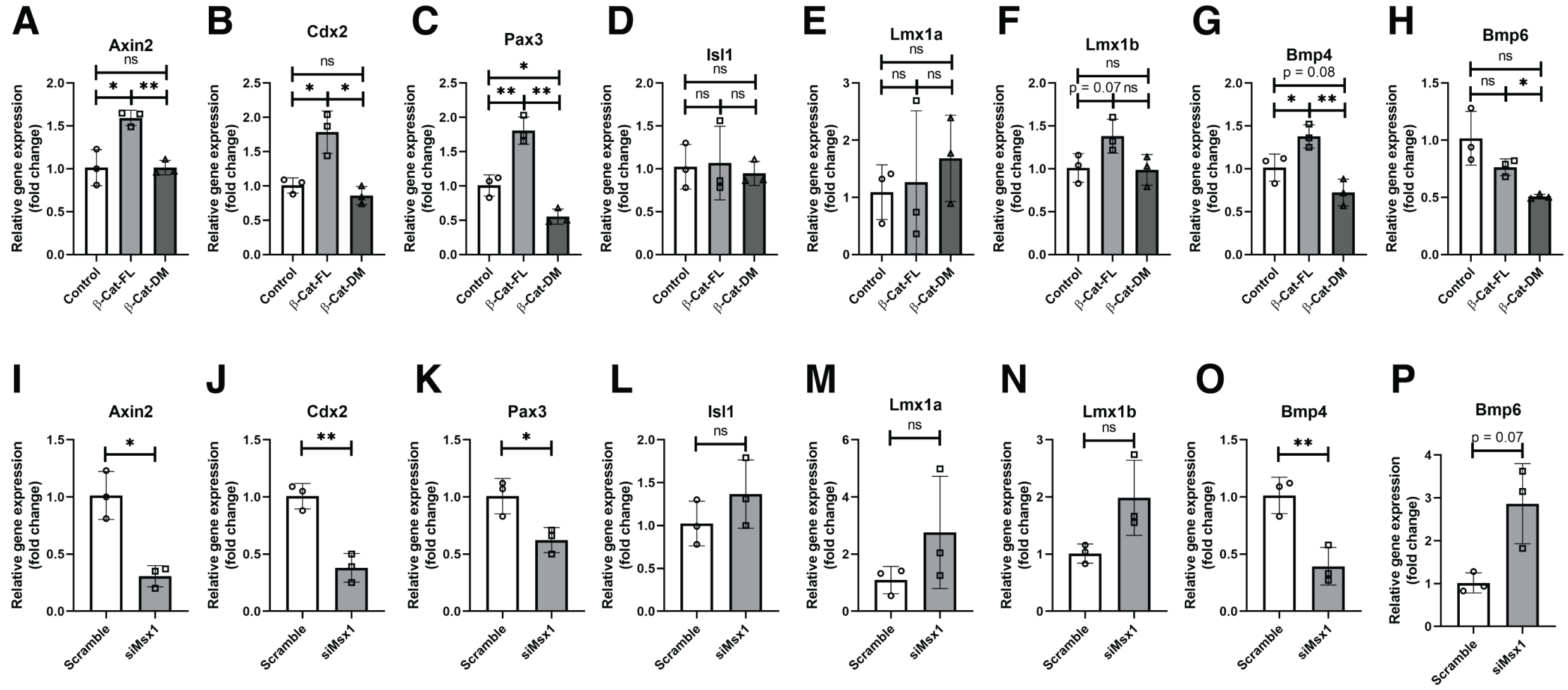

Supplementary Fig. S6. Genomic DNA alignment of mouse *Sox10* and human *SOX10* gene upstream sequences that contain a conserved Msx1 binding motif *TAATT/AATTA* (-1284 bp from the transcription start site).

```
Mouse  3376  ggaggccggatgcttgctgctc---cgtccctctgtccatccaaggcggcttctctccgg  3432
             |       |     ||||  ||   |||||||||||||||||| ||||||| ||||||||
Human  3308  gacacgacggcagatgctcgtccctcgtccctctgtccatccagggcggctcctctccgg  3367

Mouse  3433  gcggttctgaggccgggcgggcgctgttaaaaagctttttatgtgtgtgtgtgtgttaat  3492
             ||||||||||||||| ||||||||||||||||||||||||||||||||||||||  ||||
Human  3368  gcggttctgaggccgagcgggcgctgttaaaaagctttttatgtgtgtgtgtgt--taat  3425

Mouse  3493  cacatgattgccgttcacctgtatttcgaacaaagacagctcactgtttcaaggccccga  3552
             ||||||||||||||||||||||||||||||||||||||||||||||||||||||||||||
Human  3426  cacatgattgccgttcacctgtatttcgaacaaagacagctcactgtttcaaggccccga  3485

Mouse  3553  acaagagtctgggcacagggaagagaagggaggcgggggggaggagggttggcagcgccg  3612
             |||||||||||||||||||||||||||||||||||||||| |||||||||||||||||||
Human  3486  acaagagtctgggcacagggaagagaagggaggcgggggg-aggagggttggcagcgccg  3544

Mouse  3613  ggggtggcgtcgaggaaaactggggggagattgtccaaggccagcgagcgagagaaagag  3672
             ||||||||||||||||||||||||||||||||||||||||||||| ||||||||||||||
Human  3545  ggggtggcgtcgaggaaaactggggggagattgtccaaggccagcaagcgagagaaagag  3604

                                 Binding motif
Mouse  3673  aaaacccttatcaaactcctaattcactgggctctgcagccccagacacactggcccaat  3732
             |||||||||||||||||||||||||||||||||| |  ||||||||||||||||||| ||
Human  3605  aaaacccttatcaaactcctaattcactgggctccgaggccccagacacactggcccgat  3664

Mouse  3733  tgtctggctggttgtgtgtg----------------------------------------  3752
             ||||||||||||||||||||
Human  3665  tgtctggctggttgtgtgtgtgagcatgtgtgtgtgtgtgtgtgtgtgtgtgtgtgtgtg  3724

Mouse  3753  ---------c-gcaag---catgtgtgagtgtgtgttaccaatgtctgccaccccctcga  3799
                      | ||      ||      |   ||  |   |   |         ||
Human  3725  ttccagtgtctgctccccccacc-cccaacatgcctctgcctcg-tgtgactacctcatt  3782
```

Supplementary Fig. S7. Genomic DNA alignment of mouse *Atoh1* and human *ATOH1* gene upstream sequences that contain two conserved Msx1 binding motifs of *TAATT/AATTA* (-286 bp and -272 bp from the transcription start site).

```
Mouse  211   atctgagctggggccgctaggacaccagccgggagcccgcttcttctcccctcccacccc   270
                    |  | | |     |    |   | |    |||  ||  | || ||||| | ||
Human  238   CATCTGACCCGAGTCAGCTTGGGCACCAGCCGAGAGCCGGCTCCGCACCGCTCCCGCACC   297

                                                          Binding motif
Mouse  271   ccattcccctcactcaggtcgcctgggaggtgacaccctcaggagtcgaattacagcccc   330
             |||  | ||    |   | | |                   ||||||||||||||||||
Human  298   CCAGCCGCCGGGGTGGTGACACACACC--------------GGAGTCGAATTACAGCCCT   343

              Binding motif
Mouse  331   acaattaacatatgaatttccagaactgactagagggacaaggggaatttttttt-ct   389
              ||||||||||||||||                       |   | | ||
Human  344   GCAATTAACATATGAAT-------------------CTG-AC--GAATTTAAAAGAAGGA   381

Mouse  390   ttttttttttttttccagagccagagctgaactcctcggcacacagtaacttttcactgg   449
                           || ||||  |    | | ||||| |||||||  |||||||| | ||
Human  382   AAAAAAAAAAAAAACCTGAGCAGGCTTGGGAGTCCTCTGCACACAAGAACTTTTCTCGGG   441

Mouse  450   gtgtaaaaactctttgattggctcctcgcacgcgcctgcccgccccttctttgactggg   509
             ||||||||||||||||||||||| ||||||||||||||||||| |||| | ||| ||| |
Human  442   GTGTAAAAACTCTTTGATTGGCTGCTCGCACGCGCCTGCCCGCGCCCTCCATTGGCTGAG   501

Mouse  510   cagacacgcgactggcgcaaggagggggctgggaggggagccgggggagatacactggcg   569
              ||||||||||| ||||| ||||||||| |||||| ||||| ||||||||   | |||||
Human  502   AAGACACGCGACCGGCGCGAGGAGGGGGTTGGGAGAGGAGCGGGGGGAGACTGAGTGGCG   561

Mouse  570   cgtgccgctttttaaaggggcgcagcgccttcagcaacc   608
             |||||||||||||||||||||||||||||||||||||||
Human  562   CGTGCCGCTTTTTAAAGGGGCGCAGCGCCTTCAGCAACC   600
```

(The motifs AATTA/aatta at Mouse 320 and 333, and Human 332 and 346, are printed in red.)

Supplementary Fig. S8. In vitro modulation of *Wnt1* and *Wnt3a* by Msx1. (A,B) Although multiple Msx1 binding sites are found in the 5kb upstream promoter regions of both *Wnt1* and *Wnt3a* genes, *BS5* in *Wnt1* and *BS1* in *Wnt3*a are not conserved in respective human genes. (C) Chromatin immunoprecipitation demonstrates recruitments of *BS5* in *Wnt1* and *BS1* in *Wnt3*a by Msx1 antibodies. (D,E) Luciferase reporter assays demonstrate no effect or dose-dependent upregulation of Msx1 on the constructs driven by the wild-type *Wnt1_BS5* and *Wnt3a_BS1*. The constructs with rmutated motifs demonstrate no effect on *Wnt1_BS5* reporter and upregulation on *Wnt3a_BS1* of the luciferase reporter activities. ns, no statistical significance; *, $P < 0.05$; **, $P < 0.01$; ***, $P < 0.001$; ****, $P < 0.0001$.

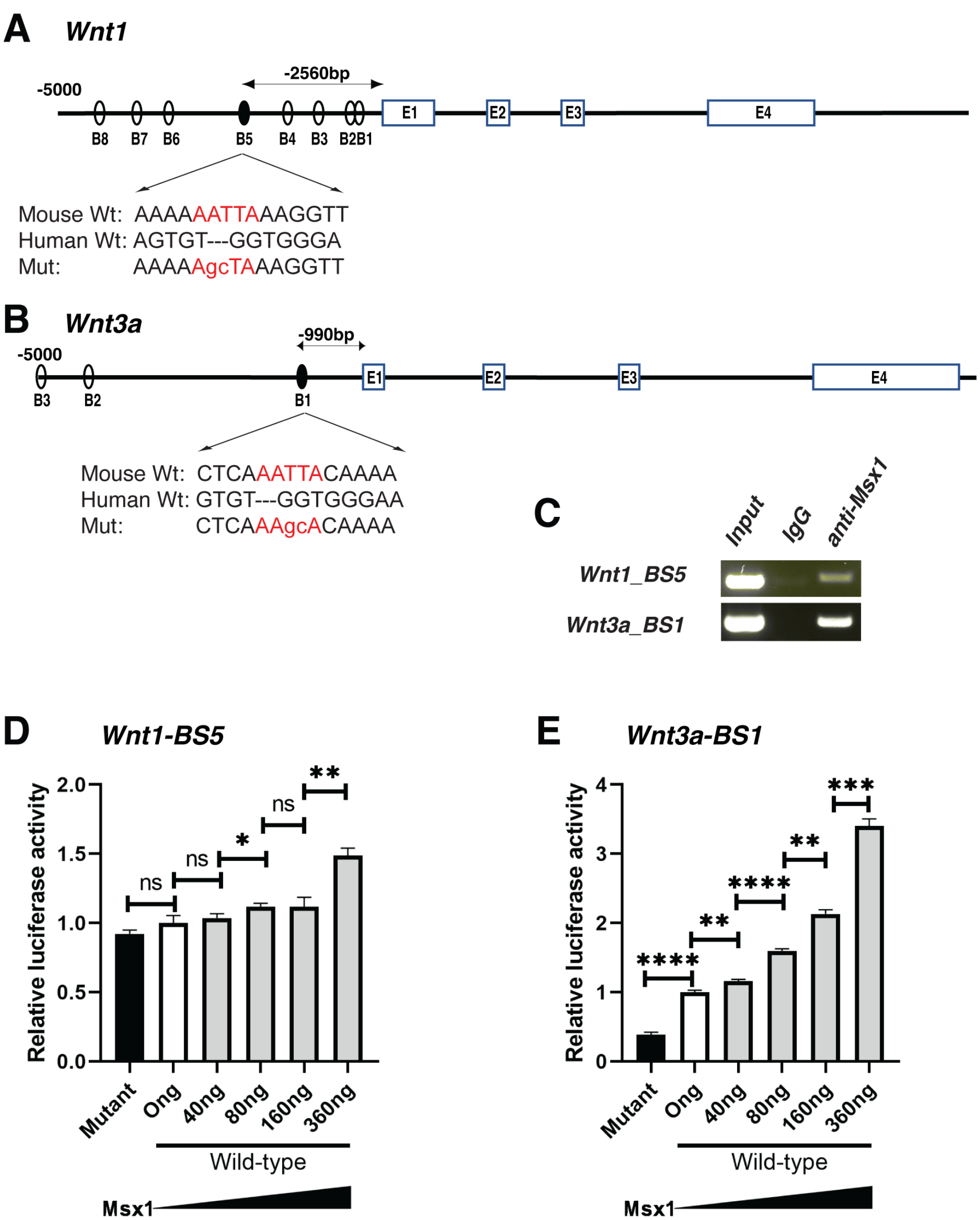

Supplementary Fig. S9. Genomic DNA alignment of mouse *Wnt1* and human *WNT1* gene downstream sequences that contain a conserved repression motif *AGAAAA* (2986 bp from the end of mouse exon 4) of Wnt/ß-catenin signaling.

```
Mouse  2279  GACTGAGTCCTGAAATATCCCAGGGGTGGCGGAAAAGCTGAGAGGACCAAGACAGCCAGC  2838
             |||| |||||||||||||||  ||||  | ||||||||||||| ||  ||| ||| | |
Human  3639  GACT-AGTCCTGAAATATCCTGGGGGGAGTGGAAAAGCTGAGAAGA--AAGGCAGGCTG-  3694

Mouse  2839  GGGGCTGCACGGTCCCTGGGGGGTCAGCAC----------CGAACACCCTGACACTGGCA  2888
                  ||||| |||||  |||||||||||           |  |||| ||| |||  |||
Human  3695  -----TGCACAGTCCCCAGGGGGTCAGCAAGCCAGCACCACACACACTCTGCCACACGCA  3750

Mouse  2889  TCTCGGAAAAACCCTGTCAGTGGTAACTGCAAATACCAGAGGGTCCAAcccccccT----  2944
              |  |||||||||||||||||||   |||||||||||  |||||||| || ||||
Human  3751  CCCTGGAAAAACCCTGTCAGTGGGG-CTGCAAATACCCTAGGGTCCAGCCTCCCCCAAAG  3796

Mouse  2945  --TCCCTACG-GCCCCAGGCAGGCTCAGTCCTTGGCTCCAG-------GGA---------  2985
               ||||  |  ||||||||| |||||||||||   ||||||       |||
Human  3797  CCTCCCCCCTCGCCCCAGGCTGGCTCAGTCCTCCTCTCCAGCGCAGAGGGAGAAAAATAA  3868

                       Repression motif
Mouse  2986  ----------AGAAAATAAGCTGTCTGGCTGTGGTGTGAGCAGGAACATCCGTGCAGAGC  3035
                       |||||||||||||| ||||||||||||||||||||| |||| ||||| ||
Human  3869  CAAAACGAGGAGAAAATAAGCTGTTTGGCTGTGGTGTGAGCAGGAATATCCATGCAGGGC  3928

Mouse  3036  TGCCCACCAACTCACCCTTACTCAGTCTTTCCACACCATGCCAGGGC--CGGGCATGAGA  3093
             ||||||||| |||||||||||||| ||||||||||||  ||| ||||  |  ||||||||
Human  3929  TGCCCACCA-CTCACCCTTACTCACTCTTTCCACACCCAGCCGGGGCGCCCAGCATGAGA  3987

Mouse  3094  CAGACCAcccccttaccccctcccccGTCTGGGCTAAAAGCAGACACACTGGGTTGGTGA  3153
             |||||||||||| | ||||||||||||||||| |||||||||||| |       ||||||
Human  3988  CAGACCACCCCCCTGCCCCCTCCCCCGTCTGGTCTAAAAGCAGACGC-------TGGTGA  4040

Mouse  3154  GAGAGCAGCAGGTTATAAATATGCCCAAGGAACTGCCGCCAACTCGCTCCCCTGTCATCC  3213
             | ||||||||||||||||||||||||| ||||||||||||||||||||||| ||||||||
Human  4041  GCGAGCAGCAGGTTATAAATATGCCCAGGGAACTGCCGCCAACTCGCTCCCTTGTCATCC  4100

Mouse  3214  CCTGGGAGGGGGTAGGGTGGGGTGACACCTAAGGAGGAGCCAGGGCTGCCCTATTCAGTG  3273
             ||||||| |||| ||||||||||| | || | |||||||||||||||||||| |||  ||
Human  4101  CCTGGGA-GGGGCAGGGTGGGGTGGCTCCCAGGGAGGAGCCAGGGCTGCCCTGTTCCCTG  4159

Mouse  3274  AGGCCCAGAGAGAAGTGGAGCCTCC  3298
             ||||||||   ||||||| || |||
Human  4160  AGGCCCAGGAGGAAGTGGGGCTTCC  4184
```

Supplementary Fig. S10. Genomic DNA alignment of mouse *Wnt3a* and human *WNT3A* intron 1 sequences that contain a nearly conserved repression motif *AGATAA* (350bp from mouse exon 1, reserve strand) of Wnt/ß-catenin signaling.

```
                   Repression motif
Mouse  347   ACCTTATCTTACACACACACACACACACACACACACACACACACACACACACACACACACACAC   408
             ||| ||||||||||||| ||||||||||||
Human  409   ACCGTATCTTACACACAAACACACACAC----------------------------------   438

Mouse  409   ACACACACACACTTTAAAACAAAGTCGGCAAGACAAGACCGAACAGCTCGGGGCAGCGCAGTT   460
                         |||||||||||||||   ||||||||| ||||||||||| ||||||  |||
Human  439   -----------GTTTAAAACAAAGTCATGAAGACAAGATCGAACAGCTCGGAGCAGCGTGGTT   491

Mouse  461   ACGTAAAGACAG   472
             ||||||||| ||
Human  492   ACGTAAAGAAAG   503
```

Supplementary Table S1. Primers used for RT-qPCR in the current study.

Gene	Forward primer (5'-->3')	Reverse primer (5'-->3')
Atoh1	GAGTGGGCTGAGGTAAAAGAG T	GGTCGGTGCTATCCAGGAG
Axin2	AAGTGTCTCTACCTCATTTTCCG	TCCAGTTTCAGTTTCTCCAGC
Bmp4	GAGCCATTCCGTAGTGCCAT	GTCCATGATTCTTGGGAGCCA
Bmp6	AGAAGGGCACTCTTTCAGGTTCCA	TCACACCACCGAGAGTCAACACAA
Cdx2	CATCACCATCAGGAGGAAAAG	CTGCGGTTCTGAAACCAAAT
Gapdh	CAACGACCCCTTCATTGACC	GGTCTCGCTCCTGGAAGATG
Isl1	GCAGCAACCCAACGACAAAA	AATTGACCAGTTGCTGAAAAGC
Lmx1a	AAATGGTAGTGGGAATGCGGGCAT	TTCTGAGGTTGCCAGGAAGCAGT
Lmx1b	AGTGTGTGTACCACTTGGGCTGTT	AGGATGCCTTGAAAGCTCTTCGCT
Mafb	TGAGCATGGGGCAAGAGCTG	CCATCCAGTACAGGTCCTCG
Msx1	CCGTGGATGCAGAGTCCC	GCTTGCGGTTGGTCTTGTGC
Msx2	ATACAGGAGCCCGGCAGATACT	AACTTGCGCTCCAAGGCTAGAA
Pax3	TGCCCTCAGTGAGTTCTATCAGC	GCTAAACCAGACCTGCACTCGGGC
Sox10	TACAAGTACCAACCTCGGCG	GGCACTCTTGTAGTGAGCCT
Wnt1	TTCGGCAAGATCGTCAACCG	GGTGATTGCGAAGATGAACGC
Wnt3a	AATTTGGAGGAATGGTCTCTCGG	CAGCAGGTCTTCACTTCACAG
Wnt5a	AGGAGTTCGTGGACGCTAGA	ACTTCTCCTTGAGGGCATCG
